# Unlocking the Potential of Palmierite Oxides: High Oxide Ion Conductivity via Induced Interstitial Defects

**DOI:** 10.1021/jacs.4c17849

**Published:** 2025-03-07

**Authors:** Dylan N. Tawse, Sacha Fop, John W. Still, Oscar J. B. Ballantyne, Clemens Ritter, Ying Zhou, James A. Dawson, Abbie C. Mclaughlin

**Affiliations:** † Advanced Centre for Energy and Sustainability (ACES), The Chemistry Department, University of Aberdeen, Aberdeen AB24 3UE, U.K.; ‡ Chemistry−School of Natural and Environmental Sciences, Newcastle University, Newcastle NE1 7RU, U.K.; § Institut Laue Langevin, 71 Avenue des Martyrs, BP 156, F-38042 Grenoble Cedex 9, France

## Abstract

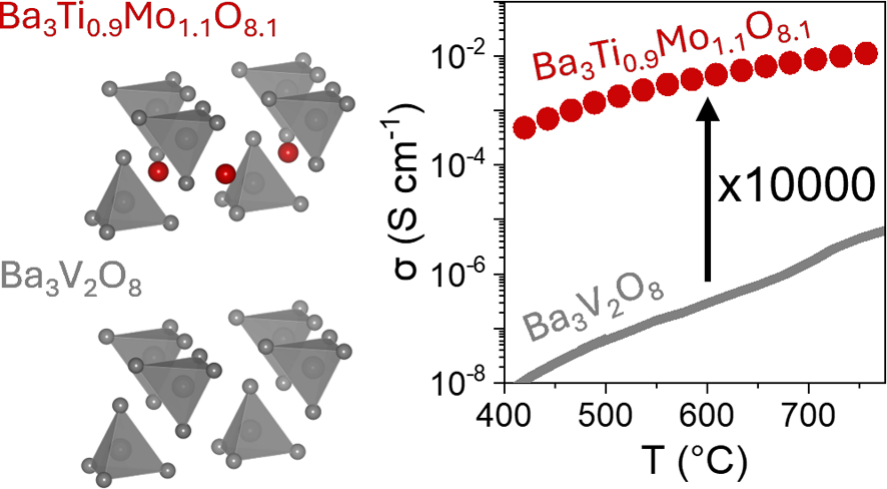

Hexagonal perovskite derivatives such as Ba_7_Nb_4_MoO_20_ and Ba_3_NbMoO_8.5_ have recently been reported to exhibit high oxide ion conductivity and have potential applications in next-generation solid oxide fuel cells. In contrast, Ba_3_V_2_O_8_ and Sr_3_V_2_O_8_ that crystallize with the structurally related palmierite structure show oxide ion conductivities orders of magnitude lower. Here we use design principles to enhance the oxide ion conductivity in palmierites. By replacing V^5+^ with two cations that are known to display flexible coordination (Mo^6+^ and Ti^4+^) and manipulating the ratio of Mo^6+^:Ti^4+^ to insert interstitial oxygen, a high oxide ion conductivity of 3.96 × 10^–3^ S cm^–1^ at 600 °C is observed in Ba_3_Ti_0.9_Mo_1.1_O_8.1_, two orders of magnitude higher than previously reported in palmierites. The oxide ion conductivity of Ba_3_Ti_0.9_Mo_1.1_O_8.1_ is also higher than that previously reported for both Ba_7_Nb_4_MoO_20_ and Ba_3_NbMoO_8.5_ at 600 °C. Introducing interstitial oxygen into the [BaO_2+*x*_] layer results in a change in the oxide ion transport from a cog-wheel type motion to an interstitialcy mechanism, demonstrating that palmierites are flexible to doping strategies via the introduction of either vacancies or oxide ion interstitials.

## Introduction

Research focusing on ionic conductors has increased considerably in recent years due to their varied applications in electrochemical hydrogen technologies, such as electrolyte materials for ceramic fuel cells and solid oxide electrolyzers.^1−7^ However, the application of many ionic conductors for everyday use is currently limited due to the high operating temperatures (600–1000 °C) required by these materials to function sufficiently.^2,8^ It is therefore imperative that new ionic conductors are discovered for the next generation of ceramic fuel cells which operate at reduced temperatures (<600 °C).

There is a strong link between the crystal structure and ionic diffusion of mobile species which is commonly related to structural disorder and defects present within the structure.^9,10^ Many crystal systems have been found to display ionic conductivity, including fluorite-type systems,^11,12^ perovskites,^13−16^ and perovskite derivatives.^17−20^

**Figure 1:**
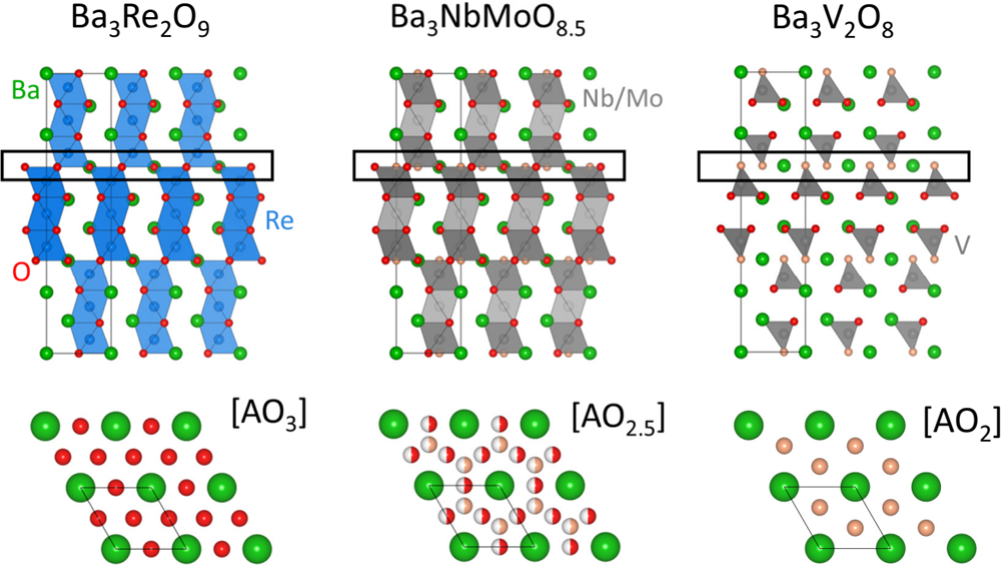
Crystal structures of related hexagonal perovskite derivative oxides. From left to right Ba_3_Re_2_O_9_ (9R), Ba_3_NbMoO_8.5_ (hybrid between the 9R and palmierite) and Ba_3_V_2_O_8_ (palmierite). The pseudocubic P-L layers of composition [BaO_2+*x*_] are highlighted across the series.

We have recently reported significant ionic conductivity in several hexagonal perovskite derivatives comprised of palmierite-like (P-L) and perovskite layers. High bulk oxide ion conductivity is observed for Ba_3_NbMoO_8.5_ (2.2 × 10^–3^ S cm^–1^ at 600 °C) and Ba_7_Nb_4_MoO_20_ (3.2 × 10^–3^ S cm^–1^ at 600 °C) under dry air.^18,19^ Under wet air, Ba_7_Nb_4_MoO_20_ exhibits proton conduction with a bulk ionic conductivity of 4.0 × 10^–3^ S cm^–1^ at 510 °C. P-L layers are present in both Ba_3_NbMoO_8.5_ and Ba_7_Nb_4_MoO_20_ in which the ionic-conducting pathways are primarily found. Ba_3_NbMoO_8.5_ crystallizes as a hybrid between the 9R perovskite and the palmierite structure which is an oxygen-deficient derivative of the 9R perovskite (Figure 1). Nb/Mo (M) cations are present on two partially occupied sites leading to a disordered cationic sublattice, while the O atoms are split across three positions, two of which are found on the P-L layer. The resulting structure is composed of variable coordination MO_*x*_ polyhedra with cationic vacancies distributed throughout.^18^ Ba_7_Nb_4_MoO_20_ is a 7H hexagonal perovskite derivative composed of 12R-like perovskite blocks and P-L layers. Partial occupancy of the M1 site on the P-L layers and M2 sites present in the 12R blocks produces cationic vacancies within both the P-L and 12R layers.^19^ Similarly to Ba_3_NbMoO_8.5_, mixed coordinate MO_*x*_ polyhedral units are observed in the P-L layers of Ba_7_Nb_4_MoO_20_ due to the partially occupied O sites.^18,19^ We have recently performed a combined experimental and computational study of the effect of applied pressure on the crystal structure and ionic conductivity of Ba_7_Nb_4_MoO_20_.^21^ The proton transport is relatively unaffected by applied pressure, but a significant reduction in oxide ion transport is observed.

Studies focusing on the palmierite oxides (Figure 1) of the formula A_3_V_2_O_8_ (A = Ba/Sr) have determined that these materials are dual oxide ion and proton conductors.^20^ Sr_3_V_2_O_8_ displays a bulk oxide ion conductivity of 3.2 × 10^–5^ S cm^–1^ at 600 °C under dry air, which is low in comparison to the structurally related Ba_3_NbMoO_8.5_ and Ba_7_Nb_4_MoO_20_. The total oxide ion conductivity of Ba_3_V_2_O_8_ under the same environment at 600 °C is significantly lower (1.8 × 10^–7^ S cm^–1^).^20^ The reduced ionic conductivity of the A_3_V_2_O_8_ phases is linked to the difference in crystal structure and oxide ion migration mechanism compared to the related Ba_3_NbMoO_8.5_ and Ba_7_Nb_4_MoO_20_ phases. Oxide ion migration in A_3_V_2_O_8_ phases occurs through a vacancy-driven mechanism in which rotation of a VO_4_ unit and oxygen-deficient VO_3_ unit allows for the movement of an oxygen atom via an intermediate V_2_O_7_ dimer; a cog-wheel type motion, as reported for La_0.8_Ba_1.2_GaO_3.9_.^20,22^

The superior oxide ion conductivities of Ba_3_NbMoO_8.5_ and Ba_7_Nb_4_MoO_20_ phases are related to the presence of extra oxygen along the pseudocubic P-L layers of composition [BaO_2+*x*_], and the existence of mixed MO*_*x*_* geometries, which essentially enable an interstitialcy oxide ion diffusion mechanism. Therefore, the stabilization of interstitial oxygen defects and the introduction of mixed cation geometries could be suitable strategies to enhance the conductivity of palmierites. A study of the A_3_V_2_O_8_ compositions demonstrated that V^5+^ cannot expand its coordination shell to accommodate an extra oxide ion due to its strong preference for tetrahedral geometry.^20,23^ Therefore, other palmierite-related compositions should be explored.

It is hypothesized that introducing metals that can exhibit multiple coordination environments and excess interstitial oxygen into the palmierite structure would allow for an increase in low-energy conduction pathways, thus increasing the ionic conductivity. The 12H hexagonal phase Ba_6_Y_2_Ti_4_O_17_ and the 21R oxygen-deficient Ba_7_Y_2_Mn_3_Ti_2_O_20_ phase both contain P-L layers of composition [BaO_2_] and [BaO_2+*x*_]. These layers are formed by the presence of TiO_4_ units and mixed TiO_4_/TiO_5_ geometries and result in mixed ionic and electronic conductivity.^24,25^ The presence of TiO_4_/TiO_5_ units in the P-L layers suggests that Ti-containing compositions are suitable candidates in the search for new palmierite-derived materials, which may exhibit stabilization of excess interstitial oxygen. Additionally, since Ti^4+^ typically displays a preference for higher coordination geometries, particularly octahedral geometry, the tendency of forming mixed coordination environments could be increased by the introduction of a cation that typically favors lower coordination tetrahedral geometry like Mo^6+^.

A previous study by Mössner and Kemmler-Sack on the Ba_3_Mo_2–*x*_Ti*_*x*_*O_9–*x*_ series determined that only phases in a narrow window around *x* = 1 could be synthesized. This study concluded that Ba_3_MoTiO_8_ crystallizes in the space group *R*3̅*m H* and is isostructural with Ba_3_V_2_O_8_, therefore displaying the palmierite structure.^26^ We synthesized the palmierite oxide derivative Ba_3_Ti_0.9_Mo_1.1_O_8.1_ with the aim of introducing interstitial oxygen into the palmierite structure *via* the substitution of Mo^6+^ for Ti^4+^. Structural elucidation of Ba_3_Ti_0.9_Mo_1.1_O_8.1_ using neutron diffraction data confirms that this approach does indeed result in interstitial oxygen on the P-L layer and high oxide ion conductivity.

## Results and Discussion

Characterization: Powder X-ray diffraction shows Ba_3_Ti_0.9_Mo_1.1_O_8.1_ is phase pure (Figure S1) and could be indexed with the space group *R*3̅*m H* (*a* = 5.94864(5) Å; *c* = 21.260(2) Å), as previously reported for the A_3_V_2_O_8_ palmierite oxides.^20^ SEM micrographs show that the grains are irregular and range in size between ∼0.5 and 4 μm (Figure S2).

**Figure 2:**
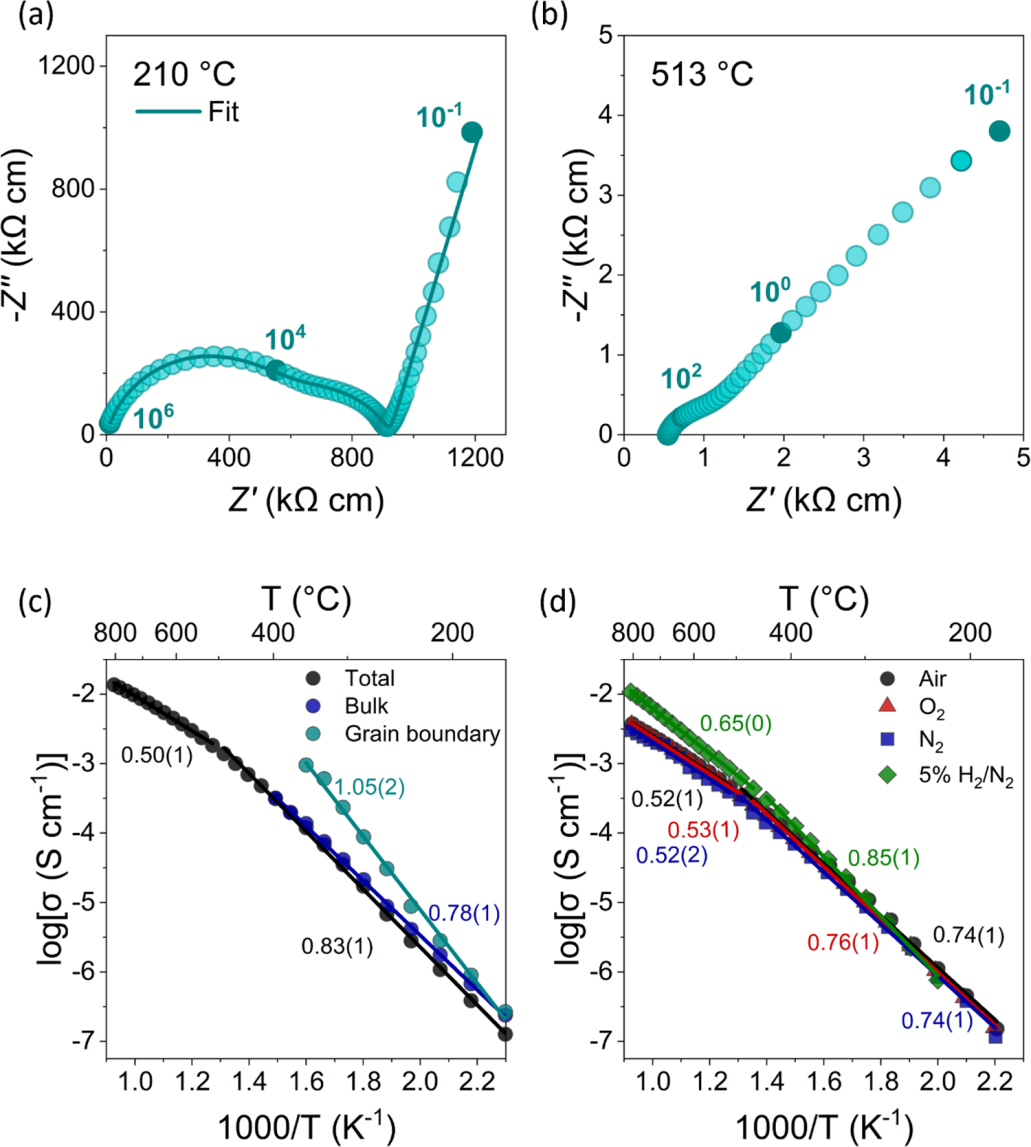
Impedance spectroscopy and conductivity of Ba_3_Ti_0.9_Mo_1.1_O_8.1_. Complex impedance plots of Ba_3_Ti_0.9_Mo_1.1_O_8.1_ at (a) 210 and (b) 513 °C recorded under dry air. The circles filled with a darker blue and corresponding numbers denote selected frequency decades in Hz. The blue line in (a) is the equivalent circuit fitting at 210 °C. (c) Total, bulk, and grain boundary conductivities of the first Ba_3_Ti_0.9_Mo_1.1_O_8.1_ sample recorded under dry air. Lines represent the fit of the data while the numbers represent the activation energies in eV. (d) Total conductivity of the second Ba_3_Ti_0.9_Mo_1.1_O_8.1_ sample recorded under dry air, dry O_2_, dry N_2_, and dry 5% H_2_/N_2_. Lines represent the fit of the data while the numbers represent the activation energies in eV.

Ionic Conductivity: AC impedance spectroscopy measurements were performed on a pellet of Ba_3_Ti_0.9_Mo_1.1_O_8.1_ with a density of ∼90.0% of the theoretical density and Pt electrodes on both surfaces. Initial measurements were performed under dry air, and subsequent measurements on a new sample were carried out under a range of dry atmospheres (air, O_2_, N_2_, and 5% H_2_/N_2_). Typical impedance spectra for Ba_3_Ti_0.9_Mo_1.1_O_8.1_ measured under dry air are shown in Figure 2a,b at various temperatures. At temperatures up to ∼260 °C, two arcs are visible in the high and intermediate regions which are related to the bulk (∼3.4–6.9 pF cm^–1^) and grain boundary (∼0.2–1.1 nF cm^–1^) responses. The grain boundary response appears to decrease with temperature with a new region arising at ∼375 °C related to the charge transfer to and from oxide ions at the sample–electrode interface with a capacitance value of ∼4.7–5.0 μF cm^–1^.^27,28^ A prominent Warburg spike is present at all temperatures in the low-frequency region (Figure S3), which indicates ionic conduction in a material with partially blocking electrodes. By ∼325 °C, the electrode response begins to dominate, and the additional responses become more difficult to distinguish (Figure S3b). The Warburg response starts to turn over at ∼560 °C to form an arc which becomes more apparent as temperature increases (Figure S3d–f).

**Figure 3:**
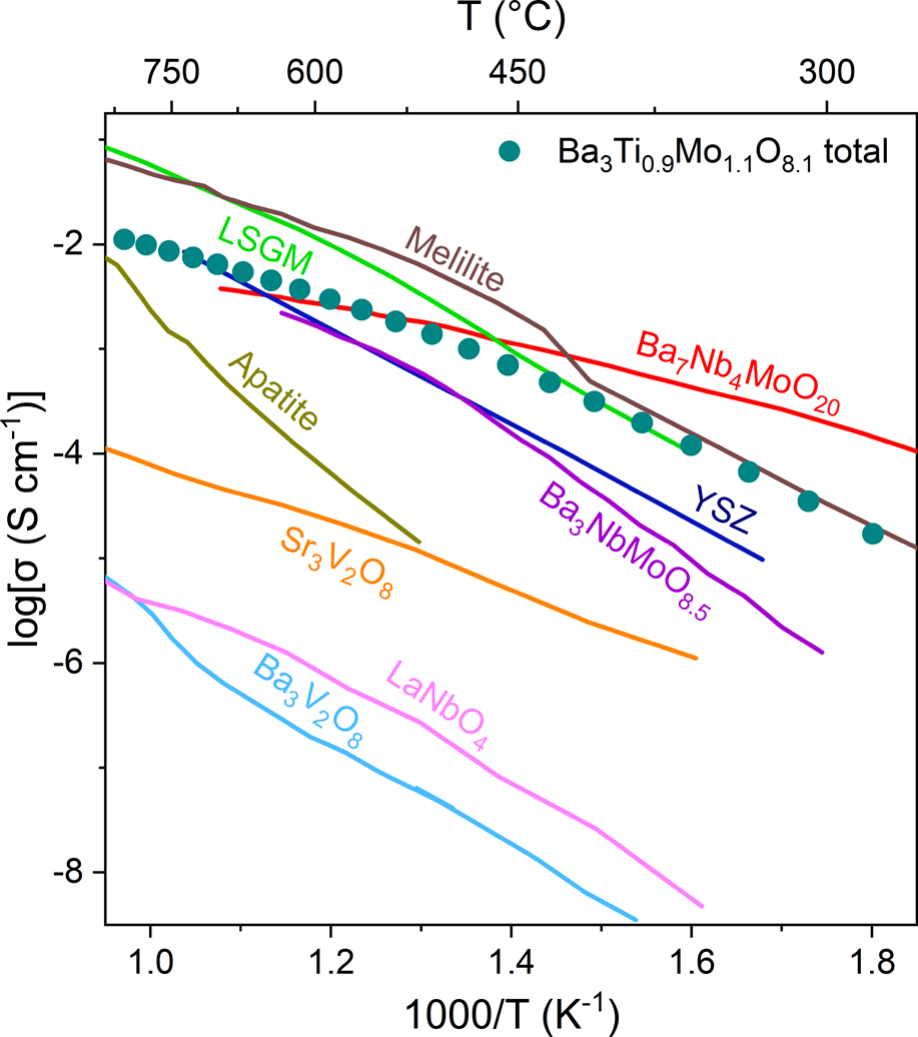
Total conductivity of Ba_3_Ti_0.9_Mo_1.1_O_8.1_ compared to total or bulk conductivities of leading ionic conductors and hexagonal perovskite derivatives. Bulk conductivities of LSGM (La_0.9_Sr_0.1_Ga_0.8_Mg_0.2_O_2.85_)^10^ Adapted from ref (10) with permission from the Royal Society of Chemistry, Copyright 2010; YSZ (Zr_0.92_Y_0.08_O_1.96_)^11^ Adapted from ref (11) with permission from Elsevier, Copyright 2006; Ba_7_Nb_4_MoO_20_^19^ Adapted from ref (19) with permission from Springer Nature, Copyright 2020; Ba_3_NbMoO_8.5_^18^ Adapted from ref (18), Copyright 2016 American Chemical Society; and Sr_3_V_2_O_8_^20^ Adapted from ref (20), Copyright 2022 American Chemical Society are shown. Total conductivities of melilite (La_1.54_Sr_0.46_Ga_3_O_7.27_)^29^ Adapted from ref (29) with permission from Springer Nature, Copyright 2008; apatite (La_10_Ge_6_O_27_)^30^ Adapted from ref (30) with permission from the Royal Society of Chemistry, Copyright 2017; LaNbO_4_^31^ Adapted from ref (31), Available under a CC BY-NC 3.0 license, Copyright 2021 Auckett, J. E., et al.; and Ba_3_V_2_O_8_^20^ Adapted from ref (20), Copyright 2022 American Chemical Society; are shown. Total conductivity of Ba_3_Ti_0.9_Mo_1.1_O_8.1_ is shown as bulk, and total conductivity converges above ∼400 °C.

Equivalent circuit fitting was performed to distinguish the individual electrical responses at each temperature. Two models were used, dependent on the electrical responses present in the data at a selected temperature. The first model (Figure S4a) was used to extract the bulk, grain boundary, and electrode responses from 162–352 °C. A representative equivalent circuit fit using this model is shown in Figure 2a of the complex impedance data collected under dry air at 210 °C. As the grain boundary response diminishes, and a charge transfer response appears upon increasing temperature a second model was employed. This second model (Figure S4b) was therefore used to extract the bulk, charge transfer region, and electrode responses at 374 and 397 °C. As temperature increases, the electrode signal dominates the impedance response, and above 397 °C only the total resistivity could be extracted from the high-frequency intercept of the electrode arc on the real impedance axis. At 600 °C, the total conductivity of Ba_3_Ti_0.9_Mo_1.1_O_8.1_ under dry air is 3.96 × 10^–3^ S cm^–1^ which is 2 orders of magnitude higher than the bulk conductivity of the palmierite Sr_3_V_2_O_8_ (3.2 × 10^–5^ S cm^–1^) and more than 4 orders of magnitude higher than the total conductivity of Ba_3_V_2_O_8_ (1.8 × 10^–7^ S cm^–1^) at 600 °C (Figure 3).^20^ The total oxide ion conductivity of Ba_3_Ti_0.9_Mo_1.1_O_8.1_ is also higher than the bulk conductivities of Ba_3_NbMoO_8.5_ (2.2 × 10^–3^ S cm^–1^) and Ba_7_Nb_4_MoO_20_ (3.2 × 10^–3^ S cm^–1^) at 600 °C under dry air.^18,19^ This demonstrates that the stabilization of interstitial oxygen defects is a suitable strategy to significantly enhance the oxide ion conductivity of palmierite oxides.

An Arrhenius plot comparing the bulk, grain boundary, and total conductivities of Ba_3_Ti_0.9_Mo_1.1_O_8.1_ is displayed in Figure 2c. As temperature increases above ∼350 °C, the grain boundary can no longer be distinguished while the bulk conductivity and total conductivity converge. At low temperatures, the bulk and grain boundary conductivities are comparable but as the bulk and total conductivities converge the grain boundary conductivity (9.60 × 10^–4^ S cm^–1^) is almost an order of magnitude higher than the bulk conductivity (1.38 × 10^–4^ S cm^–1^) by ∼350 °C. The activation energy of the total conductivity is 0.83(1) eV from ∼160 to 490 °C with a change in slope observed above ∼490 °C where the activation energy decreases to 0.50(1) eV. The bulk conductivity displays an activation energy of 0.78(1) eV while the activation energy of the grain boundary is higher at 1.05(2) eV.

Impedance spectroscopy measurements were performed under dry air, O_2_, N_2_, and 5% H_2_/N_2_ on a second Ba_3_Ti_0.9_Mo_1.1_O_8.1_ sample with a density of ∼89.4%. Figure S5 shows a typical complex impedance plot at 395 °C in each environment. At 395 °C, the bulk response corresponds to the total conductivity, as observed previously. Figure 2d shows that there is no significant change to the total conductivity upon changing the gas from dry air to O_2_ or N_2_. However, at 395 °C, the response exhibited under 5% H_2_/N_2_ corresponds to a higher total conductivity of 1.26 × 10^–4^ S cm^–1^ compared to 8.60 × 10^–5^ S cm^–1^ under dry air. This behavior suggests that *n*-type electronic conduction is present when Ba_3_Ti_0.9_Mo_1.1_O_8.1_ is heated under low *p*O_2_. A similar response is observed for Na_0.5_Bi_0.5_TiO_3_ under 5% H_2_/N_2_ in which *n*-type electronic conduction is present due to oxygen loss as a result of the partial reduction of Ti^4+^ to Ti^3+^.^13^ Hence, Ba_3_Ti_0.9_Mo_1.1_O_8.1_ exhibits a small level of *n*-type electronic conduction under 5% H_2_/N_2_ above ∼300 °C and purely oxide ion conductivity in dry air, O_2_, and N_2_ (Figure 2d). A decrease in activation energy is observed above ∼440–490 °C from ∼0.75 to ∼0.52 eV for air, O_2_, and N_2_ and from 0.85 to 0.65 eV under 5% H_2_/N_2_ (Table S1).

The impedance spectroscopy measurements under different atmospheres demonstrate that Ba_3_Ti_0.9_Mo_1.1_O_8.1_ shows predominantly oxide ion conductivity. XRD patterns collected under dry air (Figure S6), O_2_, N_2_, and 5% H_2_/N_2_ (Figure S7) show no trace of impurity phases, confirming the high stability of the material under dry conditions. Under 5% H_2_/N_2_, a color change from beige to blue is observed (Figure S8). This color change is expected to result from the reduction of Ti^4+^ to Ti^3+^ and/or Mo^6+^ to Mo^5+^ resulting in the small electronic component of the conductivity observed.

Crystal Structure: The crystal structure of Ba_3_Ti_0.9_Mo_1.1_O_8.1_ was investigated by Rietveld refinement using neutron diffraction data. Neutron diffraction measurements were performed on the high-resolution diffractometer D2B at the Institut Laue-Langevin to investigate the oxygen sublattice and determine the position of the additional oxygen atoms with data collected at 25 and 700 °C. The structural model of Ba_3_TiMoO_8_ in space group *R*3̅*m H* suggested by Mössner and Kemmler-Sack was initially employed as a starting model.^26^ The Ba atoms occupy two Wyckoff positions; Ba1 on a 3*a* site and Ba2 on a 6*c* site. Mo and Ti share the same 6*c* position and fractions were set to 0.55 and 0.45, respectively to account for the Mo:Ti ratio resulting from the stoichiometry of Ba_3_Ti_0.9_Mo_1.1_O_8.1_. Oxygen atoms are present in two Wyckoff positions; O1 at a 6*c* position and O2 at an 18*h* position. The refinement of this starting model assumed the oxygen stoichiometry was 8 and as such the occupation of the positions of the oxygen was fixed at 1. The fractional occupancies of Mo and Ti were allowed to refine with a constraint set so that the total stoichiometry of the Ti/Mo atoms was 2.

**Figure 4:**
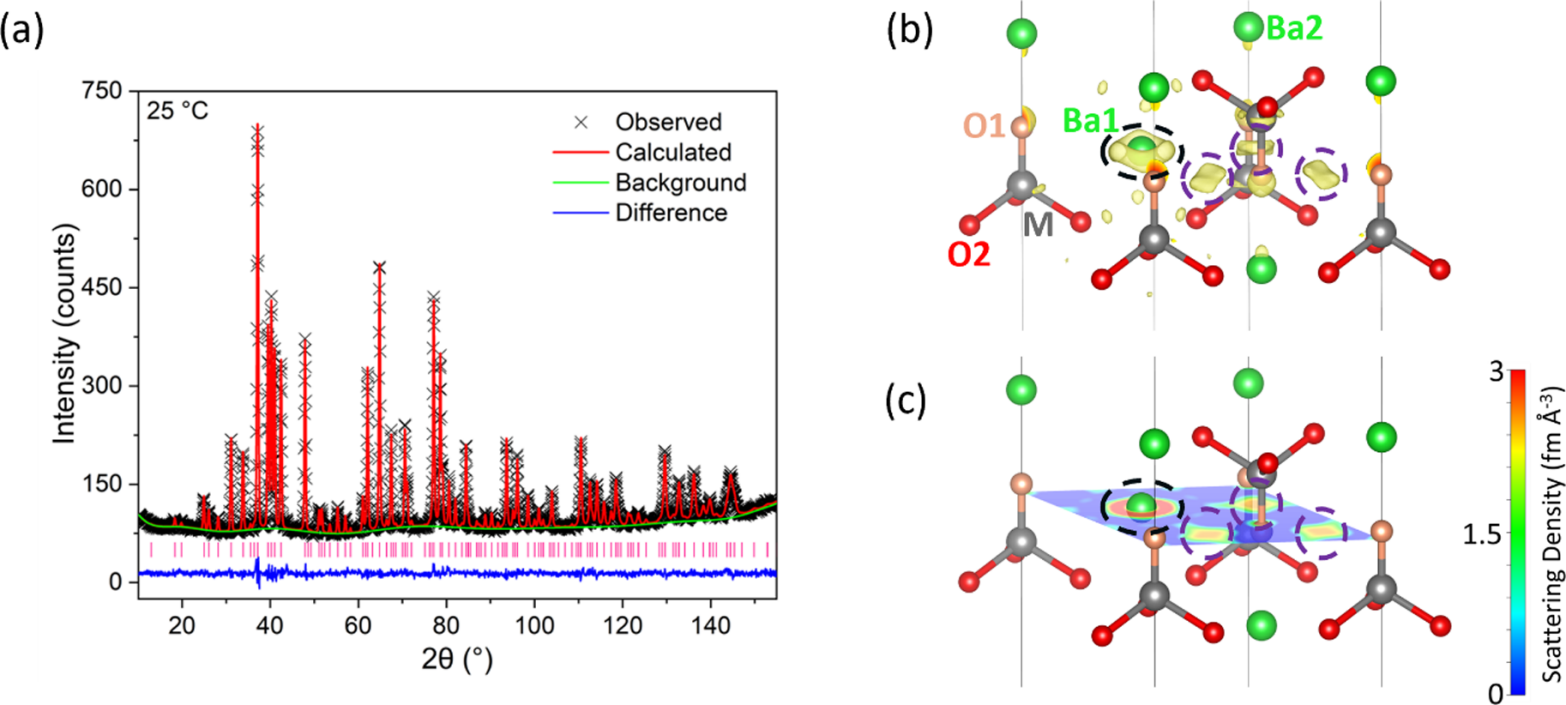
Neutron diffraction results. (a) Fitted neutron diffraction histogram for Ba_3_Ti_0.9_Mo_1.1_O_8.1_ collected on D2B at 25 °C. Black crosses show the observed data, red line the Rietveld fit, blue line the difference between the observed and calculated patterns, green line the background function, and the pink vertical bars show the reflection positions. (b) Difference Fourier map of Ba_3_Ti_0.9_Mo_1.1_O_8.1_ neutron diffraction data. Isosurfaces displaying residual scattering density and (c) map at *z* ≈ 0.667 viewed along the [001] direction. Neutron scattering length density shown in the range of 0–3 fm Å^–3^. Residual scattering density is observed around the Ba1 site (circled in black) and close to the O1 site (circled in purple).

A difference Fourier map was calculated to determine the position of the additional oxygen (Figure 4). The difference Fourier map displayed two distinct areas of missing scattering density. The first area surrounds the Ba1 position suggesting that this site is highly disordered along the *ab* plane and the atomic displacement parameters for Ba1 should be modeled anisotropically. The second area of residual scattering density corresponds to the additional oxygen and is located at interstitial sites close to the O1 position.

The missing scattering density around the O1 site could be assigned to the atomic position of (0.544, 0.623, 0.007) split Wyckoff site 36*i*. Thus, it was chosen as the starting position for the O3 interstitial site and added to the model. The occupancy of O3 was set to 0.00833 to ensure an O stoichiometry of 8.1. Fractional occupancies of the metal atoms and O1/O3 sites were refined. The atomic displacement parameters for the Ba1 site were modeled anisotropically due to the missing scattering density observed on the difference Fourier map. The atomic displacement parameters for all other atoms were also modeled anisotropically, except the O3 site, which was modeled isotropically. The resulting anisotropic displacement parameters for the O1 site were high (*U*_11_ = *U*_22_ = 0.0667(14) Å^2^, and *U*_33_ = 0.0117(13) Å^2^). To resolve the high displacement parameters, the O1 position was modeled as a split site (Wyckoff site 36*i*) with a starting position of (0.05, 0, 0) and allowed to refine freely. The atomic displacement parameter for O1 was therefore refined isotropically and produced a more reasonable value of *U*_*iso*_ = 0.016(1) Å^2^.

The statistical parameters and Rietveld fit improved with the addition of the O3 atom, splitting O1 onto the 36*i* site, and modeling the Ba1 atomic displacement parameters anisotropically from χ^*2*^ = 2.340, *R*_p_ = 2.46%, *R*_wp_ = 3.17% to χ^*2*^ = 1.857, *R*_p_ = 2.18%, *R*_wp_ = 2.82% (Figure 4a and Table S2).

**Figure 5:**
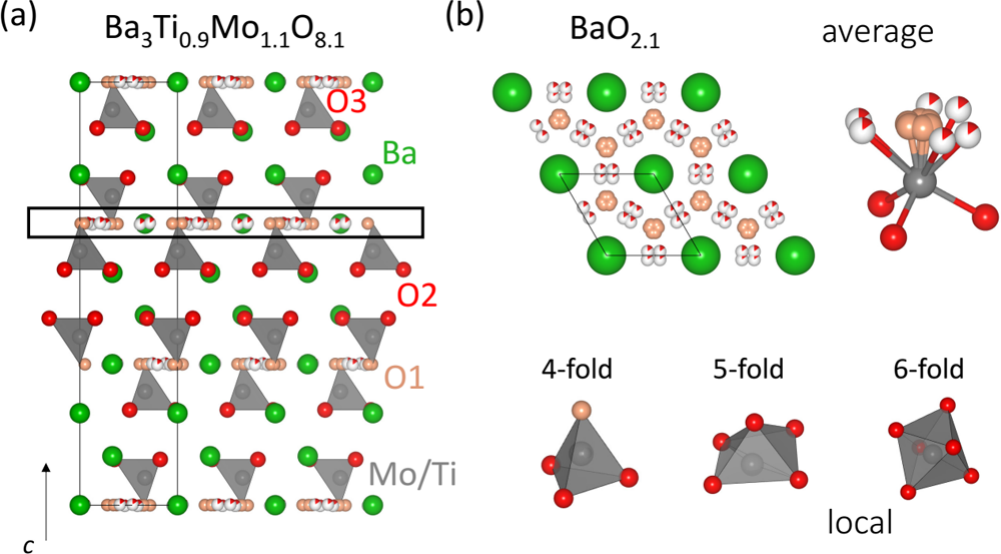
Average crystal structure of Ba_3_Ti_0.9_Mo_1.1_O_8.1_. (a) Crystal structure of Ba_3_Ti_0.9_Mo_1.1_O_8.1_, showing the isolated tetrahedral MO_4_ units with interstitial oxygen defects. (b) [BaO_2.1_] layer and representation of the average and local metal coordination in Ba_3_Ti_0.9_Mo_1.1_O_8.1_. The [BaO_2.1_] layer is composed by partially occupied and split O1 and O3 positions, which leads to the formation of disordered metal polyhedra with average composition MO_*x*_, resulting in 4-, 5-, and 6-fold coordination units on the local scale.

The results from the Rietveld refinement show that the O3 atoms occupy a split site found within the same layer as the atoms of the O1 and Ba1 sites. The O2 site is fully occupied, while the O1 and O3 sites are partially occupied. Due to the short distance between the O1 and O3 sites, these sites cannot be occupied at the same time, similar to the oxygen positions found within the primary conduction layers of Ba_3_NbMoO_8.5_ and Ba_7_Nb_4_MoO_20_.^18,19^ The O3 position would allow for the formation of local 4-, 5-, and 6-fold coordinated polyhedral units as opposed to exclusively tetrahedral units which are observed for other palmierite materials.^20^ Static disorder of the O atoms within the P-L layer is evidenced by the need to model both the O1 and the O3 positions as split sites. The highly anisotropic behavior exhibited by the Ba1 site (demonstrated by the *U*_*11*_ and *U*_*22*_ parameters) is likely related to the disorder displayed by the O1 and O3 sites along this layer. The Mo and Ti sites are both partially occupied and refinement of these fractions results in a phase with the stoichiometry of Ba_3_Ti_0.9_Mo_1.1_O_8.1_ which matches the nominal composition. The average crystal structure of Ba_3_Ti_0.9_Mo_1.1_O_8.1_ is shown in Figure 5a. The crystal structure of Ba_3_Ti_0.9_Mo_1.1_O_8.1_ hence contains [BaO_2.1_] layers, and the cationic sublattice consists of isolated polyhedral units of mixed coordination separated by octahedral vacancies.

This is the first time that interstitial oxygen has been stabilized in a palmierite consisting of predominantly isolated MO_4_ tetrahedra. Ba_3_VWO_8.5_ crystallizes in the *R*3̅*m H* space group and has a similar crystal structure to Ba_3_Ti_0.9_Mo_1.1_O_8.1_.^32,33^ However, Ba_3_VWO_8.5_ contains a lot more octahedra within the P-L layer (the fractional occupancy of the octahedral oxygen site is 0.361 and 0.017 for Ba_3_VWO_8.5_ and Ba_3_Ti_0.9_Mo_1.1_O_8.1_, respectively). The bulk oxide ion conductivity (5 × 10^–5^ S cm^–1^ at 600 °C) is also significantly lower than that of Ba_3_Ti_0.9_Mo_1.1_O_8.1_. This would suggest that a high fraction of MO_4_ tetrahedra and a small concentration of interstitial sites are required to achieve a high oxide ionic conductivity in palmierites.

Neutron diffraction data collected at 700 °C show no significant changes in the crystal structure (Figure S10). Large atomic displacement parameters are observed for Ba1, O1, and O3 resulting from the high temperature at which these data were collected and because of the disorder of the O1 and O3 positions caused by the various coordination environments in this layer. The large static disorder on the P-L layer suggests that a variety of local arrangements (i.e., 4-, 5-, and 6-fold coordination polyhedra) are present (Figure 5b).

**Figure 6:**
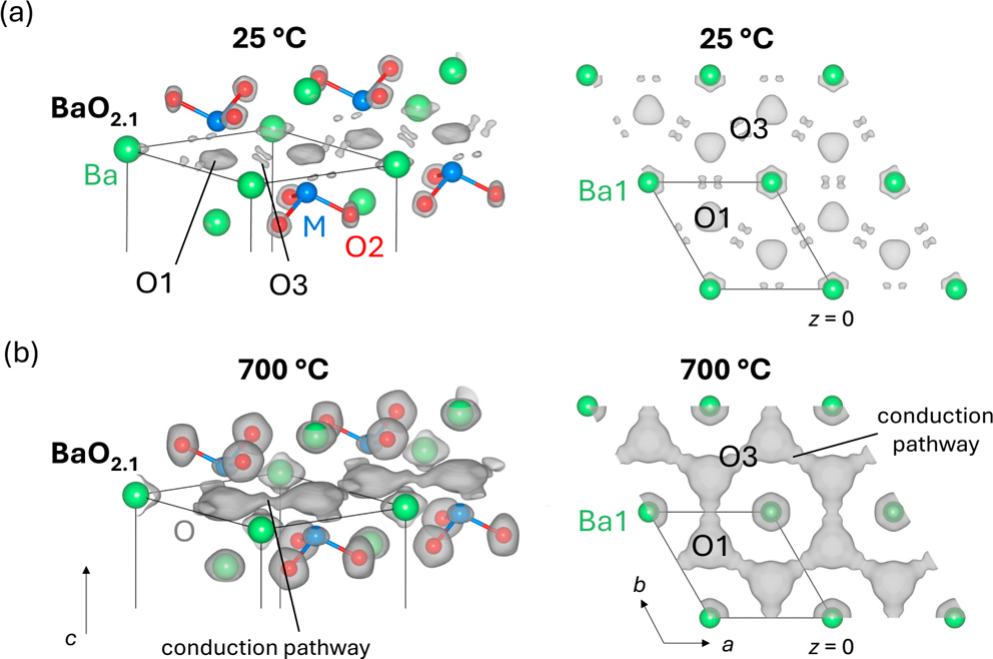
MEM analysis of the oxide ion disorder and conduction pathways in Ba_3_Ti_0.9_Mo_1.1_O_8.1_. Nuclear scattering density distribution at 25 °C (a) and 700 °C (b) reconstructed via maximum entropy (MEM) analysis. Connectivity between the oxygen O1 and O3 isosurfaces at 700 °C identifies long-range oxide ion conduction pathways along the *ab* plane. Gray isosurfaces are drawn at 1 fm Å^–3^. The structural model is superimposed to the nuclear scattering density; O1 and O3 atoms are omitted for clarity.

Conduction pathways: Maximum entropy (MEM) analysis of the neutron diffraction data was employed to study the oxide ion conduction pathways. MEM minimizes biases imposed by the structural model and provides an approach suitable for the study of aspects that go beyond the independent atom approximation, therefore it is particularly suitable for the identification of disorder and ionic conduction pathways.^34−37^ MEM analysis of the neutron data collected at 25 °C clearly shows a neutron scattering density adjacent to the apical tetrahedral oxygen O1 (Figure 6a), thus confirming the existence of interstitial oxygen O3 defects. The shapes of the O1, O3, and Ba1 scattering densities confirm the adopted split oxygen model, as well as the large static disorder within the [BaO_2.1_] layer. MEM analysis of the neutron diffraction data collected at 700 °C shows connected distributions of oxide ions, and direct evidence of oxide ion diffusion on the O1–O3 pathways along the [BaO_2.1_] layer (Figure 6b). The static oxygen disorder becomes dynamic at high temperatures, thus creating low-energy conduction pathways. Similarly to Ba_3_NbMoO_8.5_, the oxide ion conduction pathways in Ba_3_Ti_0.9_Mo_1.1_O_8.1_ are 2-dimensional on the *ab* plane,^37^ with an interstitialcy mechanism. For comparison, MEM analysis of the neutron diffraction data of Sr_3_V_2_O_8_ at 800 °C does not show any oxide ion connectivity on the [SrO_2_] plane (Figure S11). The introduction of a small concentration of interstitial oxygen defects is crucial in enabling long-range two-dimensional ionic diffusion on the [BaO_2+*x*_] layer and high oxide ion conductivity.

**Figure 7:**
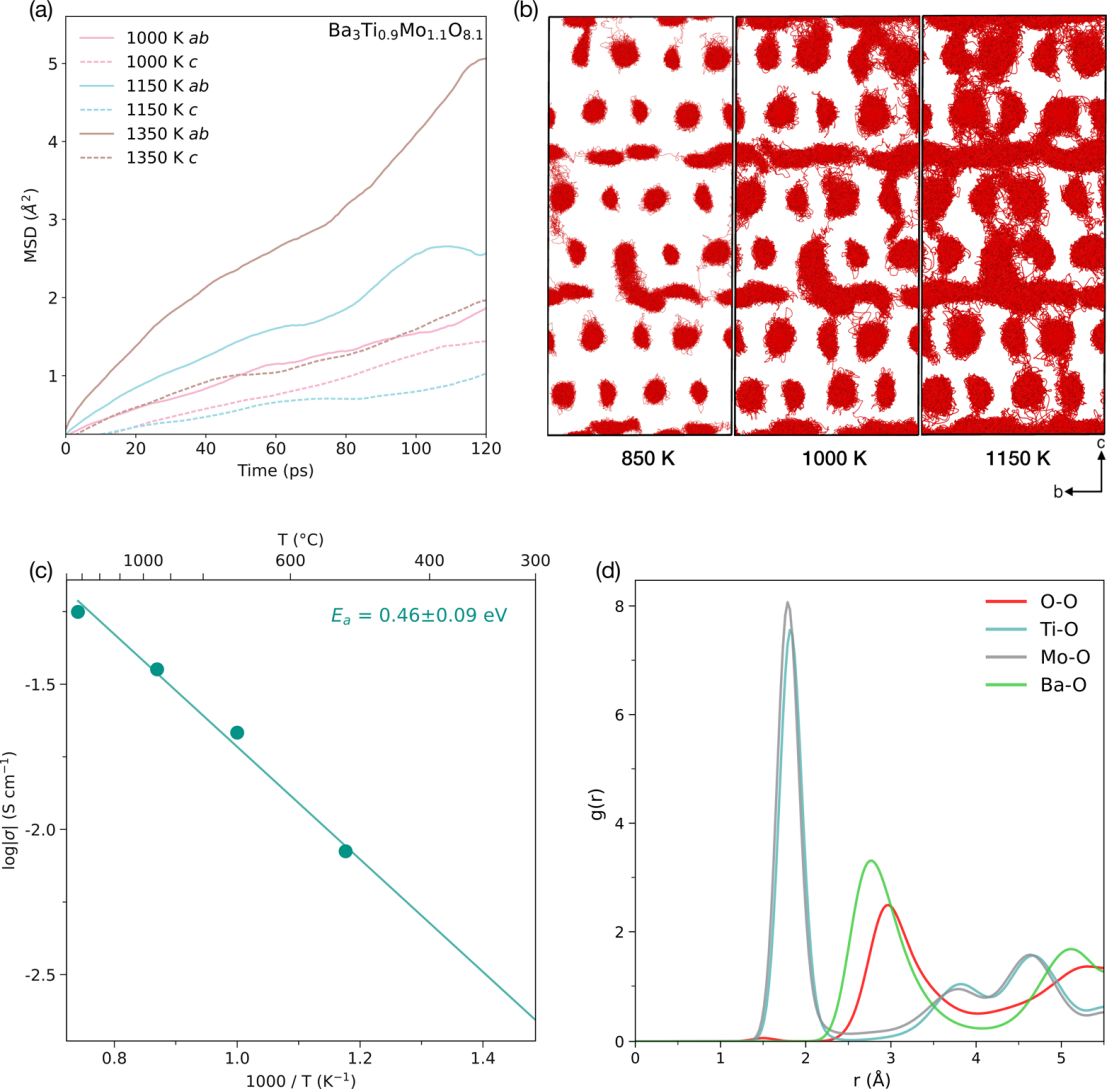
AIMD simulations of oxide ion disorder and conduction pathways in Ba_3_Ti_0.9_Mo_1.1_O_8.1_. (a) MSD plots of oxide ions in the *ab* plane and *c* direction at 1000, 1150, and 1350 K. (b) Oxide ion density plots showing the diffusion pathways at 850, 1000, and 1150 K. (c) Arrhenius plot of oxide ion conductivity. (d) RDFs of O–O, Ti–O, Mo–O, and Ba–O pairs at 850 K.

Ab initio molecular dynamics simulations at different temperatures were carried out to further explore oxide ion conduction and its mechanisms in Ba_3_Ti_0.9_Mo_1.1_O_8.1_. The calculated MSDs of the oxide ions at 1000, 1150, and 1350 K are shown in Figure 7a for both diffusion in the *ab* plane and *c* direction. In agreement with the MEM analysis above, oxide ion diffusion primarily occurs in the *ab* plane through the [BaO_2.1_] layer via the interstitial O3 site. At the higher temperatures of 1150 and 1350 K, the oxide ion diffusion in the *ab* plane is more than double that in the *c* direction. Nevertheless, as illustrated by the oxide ion density plots in Figure 7b, there is still reasonable oxide ion diffusion in the *c* direction, meaning that Ba_3_Ti_0.9_Mo_1.1_O_8.1_ is not an entirely 2D ion conductor.

At 850 K, oxide ion diffusion is limited, but there are still distinct hops occurring, particularly across the *ab* plane. When the temperature is raised to 1000 K, clear patterns begin to emerge, and diffusion along the [BaO_2.1_] layers is very evident. At 1350 K, hops between MO_4_ and MO_5_ polyhedra become frequent and the oxide ion conduction pathways connect the individual polyhedra across the *ab* plane through the interstitial oxygen O3 site. This diverges from the traditional cog-wheel type mechanism observed previously in palmierite oxides and similar material families,^20,38^ and appears to markedly enhance conductivity compared to vacancy-driven conduction pathways, such as that observed in Sr_3_V_2_O_8_. This is further supported by the neutron diffraction data, which show the migration of the ions of O1–O3 along the [BaO_2.1_] layers. The simulations reveal a conduction pathway dominated by interstitials, yielding greater conductivity in the structure without the need for oxygen vacancies (necessary for the prototypical cog-wheel mechanism of palmierite oxides).

Using the calculated diffusion coefficients for oxygen, the Arrhenius behavior of the oxide ion conductivity in Ba_3_Ti_0.9_Mo_1.1_O_8.1_ is plotted in Figure 7c. The calculated activation energy of 0.46 ± 0.09 eV is in excellent agreement with the experimental result of 0.50 eV (in dry air).

At 850 K (576.85 °C), the calculated conductivity of Ba_3_Ti_0.9_Mo_1.1_O_8.1_ is 7.89 × 10^–3^ S cm^–1^, which is in close agreement with the experimental value for the total conductivity (3.96 × 10^–3^ S cm^–1^). In comparison, in our previous work, we were unable to accurately calculate the oxide conductivity of Sr_3_V_2_O_8_,^20^ from AIMD simulations at similar temperatures due to it being significantly inferior (around 2 orders of magnitude) to that of Ba_3_Ti_0.9_Mo_1.1_O_8.1_. This marked difference in conductivities reinforces the mechanistic differences between oxide ion transport in the two palmierite structures. While the oxide ion transport mechanism of Sr_3_V_2_O_8_ is vacancy-driven, the mechanism of ion transport in Ba_3_Ti_0.9_Mo_1.1_O_8.1_ is through the interstitial oxygen in the [BaO_2.1_] layers, which appears to proceed more efficiently than the vacancy mechanism, resulting in elevated oxide ion conductivity.

The radial distribution functions (RDFs) of specific ion pairs in Ba_3_Ti_0.9_Mo_1.1_O_8.1_ were analyzed during the AIMD simulations at 850 K to elucidate notable structural motifs that may affect ion transport in Ba_3_Ti_0.9_Mo_1.1_O_8.1_ (Figure 7d). The relative magnitudes and positions of the three metal-O peaks can be attributed to the charges and sizes of the metal ions. The integrated RDFs suggest that the coordination environments of the Mo^6+^ and Ti^4+^ species are almost identical (Figure S12). A broad peak in the O–O RDF can be observed at ∼2.9 Å which fluctuates past 5.5 Å, indicating a degree of inherent disorder in the system that can be attributed to oxygen diffusion along the P-L layers.

## Conclusions

The introduction of oxygen interstitials into the palmierite Ba_3_TiMoO_8_ via compositional engineering results in a remarkable increase in oxide ion conductivity compared to previously reported palmierites.^20^ Introducing interstitial oxygen into the [BaO_2+*x*_] layer results in a change in the oxide ion transport from a cog-wheel type motion (as reported for A_3_V_2_O_8_ (A = Ba, Sr))^20^ to an interstitialcy mechanism for Ba_3_Ti_0.9_Mo_1.1_O_8.1_. This results in an increase in the total ionic conductivity by more than 3 orders of magnitude. The total ionic conductivities at 600 °C of Ba_3_Ti_0.9_Mo_1.1_O_8.1_, Sr_3_V_2_O_8_ and Ba_3_V_2_O_8_ are 3.96 × 10^–3^, 2.0 × 10^–6^, and 1.8 × 10^–7^ S cm^–1^, respectively. This study highlights how palmierite oxides are a versatile family able to accommodate different types of defects and how the different defects can lead to different mechanisms of ionic transport. Interstitial oxide ion conductors are much less common than vacancy-mediated conductors and are a promising route to reduced-temperature oxide ion electrolytes and electrolyzers.^39^ The radial distribution function results suggest that the coordination environments of Ti^4+^ and Mo^6+^ are almost identical. Furthermore, it is not possible to synthesize Ba_3_TiMoO_8_ as a pure phase. These results would hence suggest that the presence of interstitial oxygen in the [BaO_2.1_] layers results in mixed coordination Ti/MoO*_*x*_* polyhedra (4-, 5-, and 6-fold coordination polyhedra) and a more stable coordination environment for Ti^4+^. The results described in this paper define design rules for achieving high oxide ionic conductivity in palmierites for the first time. Within the P-L layer, transition metals are needed that are stable in different coordination geometries alongside a small fraction of interstitial oxygen (∼0.1). Doping studies to introduce interstitial oxide ions into Mo^6+^ containing palmierites such as KBa_2_Nb_1–*x*_Mo_1+*x*_O_8+*x*/2_ are warranted.^40^

## Experimental Section

Synthesis and Characterization: Ba_3_Ti_0.9_Mo_1.1_O_8.1_ was prepared by the solid-state reaction of stoichiometric amounts of BaCO_3_ (99.999%, Aldrich) and TiO_2_ (Anatase, 99.9%, Aldrich) with MoO_3_ (≥99.5%, Aldrich) in excess of 1.5% by weight. The starting materials were ground, pressed into a 13 mm pellet, and transferred to an alumina crucible to be heated at 1000 °C for 10 h before cooling to room temperature at 5 °C min^–1^. The sample was reground, pelleted, and reheated until a phase pure product was obtained. Laboratory X-ray diffraction was performed using a PANalytical Empyrean diffractometer equipped with a Cu Kα tube and a Johansson monochromator to determine sample purity. Data were collected in the range 5 < 2θ < 120° with a step size of 0.013°.

Stability: Samples were annealed under dry air for 10 h at 400, 600, 800, and 1000 °C. X-ray diffraction patterns were collected after each temperature to investigate the phase stability under dry conditions over this temperature range. In addition, phase stability under oxidizing and reducing conditions was studied by exposing samples to O_2_, N_2_, and 5% H_2_/N_2_ which had been flown over a commercial desiccant (Drierite, Aldrich) for 10 h at 400 and 600 °C followed by the collection of X-ray diffraction patterns after each annealing step. Scanning electron microscopy (SEM) images were recorded at the ACEMAC facility at the University of Aberdeen with a Carl Zeiss Gemini SEM 300 instrument with an XMax 80 detector and an AZtecHK EBSD analysis system with a Nordlys Nano EBSD camera (Oxford Instruments Ltd.). Samples were mounted on a stub and coated with a thin layer of carbon.

Thermogravimetric analysis (TGA) was carried out using a Mettler Toledo TGA 2 coupled with a Hiden Quadrupole Mass Spectrometer (MS). The sample was heated from 25 to 300 °C at a rate of 10 °C min^–1^ under dry air and held at 300 °C for 2 h before cooling back to 25 °C at 10 °C min^–1^ to drive off surface water. Following this step, the sample was heated from 25 to 1000 °C at a rate of 10 °C min^–1^ measuring the mass loss and tracking the MS for 44 amu (CO_2_), 32 amu (O_2_), and 18 amu (H_2_O).

Impedance Spectroscopy: A pellet of 10 mm diameter and ∼1.5 mm thickness was prepared from a powder sample of Ba_3_Ti_0.9_Mo_1.1_O_8.1_ sintered at 1000 °C for 10 or 20 h to achieve ∼90% of theoretical density. Pt paste was applied to both surfaces of the pellet and fired at 900 °C for 10 min to create Pt electrodes.

AC Impedance spectroscopy measurements were performed over a frequency range of 0.1 Hz to 1 MHz using a Solartron 1260 impedance analyzer with an applied alternating voltage of 0.1 V. Measurements were carried out under air, O_2_, N_2_, and 5% H_2_/N_2_ (all gases were flown through a Dreschel bottle filled with Drierite) over a temperature range of ∼160–800 °C. The ZView software was used to analyze the collected impedance data which was corrected using the geometrical factor. Equivalent circuit fitting analysis was performed on data collected under dry air to extract individual electrical responses, while total resistivity values under all conditions were extracted from the high-frequency intercept of the arcs on the real impedance axis.

Structural Analysis: Variable temperature neutron diffraction data were collected at 25 and 700 °C under vacuum on the high-resolution diffractometer D2B at the Institut Laue-Langevin (ILL) in Grenoble, France. A ∼3 g sample of Ba_3_Ti_0.9_Mo_1.1_O_8.1_ was loaded into an 8 mm vanadium can and heated to the desired temperature and the data were recorded at λ = 1.59432 Å for ∼3 h at 25 °C and ∼2 h at 700 °C. Rietveld refinements were performed using the GSAS/EXPGUI software package.^41,42^ An initial Rietveld refinement was performed using high-resolution laboratory X-ray diffraction data using the palmierite structure previously reported for Sr_3_V_2_O_8_ as a starting model.^43^ Maximum entropy (MEM) analysis was performed with the software Dysnomia,^34^ employing the structure factors obtained by Rietveld refinement of the neutron diffraction data. Neutron scattering density distributions at 25 and 700 °C were reconstructed by MEM calculations with the unit cell divided into 84 × 84 × 300 pixels.

Computational Methods: The Vienna ab initio simulation package (VASP)^44^ was used to carry out density functional theory (DFT) simulations. The projector augmented wave (PAW) method^45^ with the Perdew–Burke–Ernzerhof revised for solids (PBEsol)^46^ exchange-correlation functional alongside the generalized gradient approximation (GGA) was used for all simulations. A 2 × 2 × 1 supercell consisting of 157 atoms was used to account for the disordered Ba_3_Ti_0.9_Mo_1.1_O_8.1_ structure. The calculated lattice parameters of *a* = 5.96 Å and *c* = 21.32 Å are in excellent agreement with the XRD and neutron values of *a* = 5.95 and *c* = 21.26 Å. This supercell was used as the starting point for all DFT simulations. Screening of the possible Ti/Mo ordering revealed no strongly preferential configuration and therefore a random Ti/Mo ordering was utilized (Figure S13). The constructed supercell has an overall stoichiometry of Ba_3_Ti_0.92_Mo_1.08_O_8.08_, which is in good agreement with the experimental composition. Geometry optimizations were carried out using a plane-wave cutoff of 520 eV and sampling of the k-space at the Γ-point only. Ionic relaxation proceeded until the forces on the ions were less than 0.01 eV Å^–1^.

The ab initio molecular dynamics (AIMD) simulations were carried out using a plane-wave cutoff energy of 50 eV and *k*-space was again sampled at the Γ-point only due to the size of the supercell. The simulations were continued to 150 ps from 700 to 1350 K at intervals of 150 K. A simulation time of 150 ps was used as a reasonable compromise between the statistical significance and computational expense. At this time scale, a confident estimate of the oxygen diffusion coefficient can be extracted from the mean squared displacement over time. All simulations used the NVT ensemble with the Nosé–Hoover thermostat^47^ and a time step of 2 fs to account for the movement of the oxide ions. Self-diffusion data for the oxide ions were calculated according to
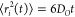
1where ⟨*r*_*i*_^2^(*t*)⟩ is the mean squared displacement, *D*_O_ is the oxygen diffusion coefficient, and *t* is time. The Python Materials Genomics (pymatgen) library^48^ was used to calculate the mean squared displacement of oxide ions over time to extract diffusion coefficients at varying temperatures. The Arrhenius behavior and activation energies were subsequently calculated.

## Supporting Information Available

X-ray diffraction patterns, additional complex impedance plots, equivalent circuit models, table of activation energies, TGA-MS and stability data, Rietveld refinement tables and fitting, table of bond lengths and angles, and additional MEM results (PDF)

## References

[ref1] Haile S. M. Fuel Cell Materials and Components. Acta Mater. 2003, 51 (19), 5981–6000. 10.1016/j.actamat.2003.08.004.

[ref2] Steele B. C. H.; Heinzel A. Materials for Fuel-Cell Technologies. Nature 2001, 414, 345–352. 10.1038/35104620. 11713541

[ref3] Coduri M.; Karlsson M.; Malavasi L. Structure-Property Correlation in Oxide-Ion and Proton Conductors for Clean Energy Applications: Recent Experimental and Computational Advancements. J. Mater. Chem. A 2022, 10 (10), 5052–5110. 10.1039/D1TA10326A.

[ref4] Shim J. H. Ceramics Breakthrough. Nat. Energy 2018, 3 (3), 168–169. 10.1038/s41560-018-0110-7.

[ref5] Brisse A.; Schefold J.; Zahid M. High Temperature Water Electrolysis in Solid Oxide Cells. Int. J. Hydrog. Energy 2008, 33 (20), 5375–5382. 10.1016/j.ijhydene.2008.07.120.

[ref6] Laguna-Bercero M. A. Recent Advances in High Temperature Electrolysis Using Solid Oxide Fuel Cells: A Review. J. Power Sources 2012, 203, 4–16. 10.1016/j.jpowsour.2011.12.019.

[ref7] Jacobson A. J. Materials for Solid Oxide Fuel Cells. Chem. Mater. 2010, 22 (3), 660–674. 10.1021/cm902640j.

[ref8] Wachsman E. D.; Taek Lee K. Lowering the Temperature of Solid Oxide Fuel Cells. Science 2011, 334 (6058), 935–939. 10.1126/science.1204090. 22096189

[ref9] Fop S.; McCombie K. S.; Wildman E. J.; Skakle J. M. S.; Mclaughlin A. C. Hexagonal Perovskite Derivatives: A New Direction in the Design of Oxide Ion Conducting Materials. Chem. Commun. 2019, 55 (15), 2127–2137. 10.1039/C8CC09534E. 30676598

[ref10] Malavasi L.; Fisher C. A. J.; Islam M. S. Oxide-Ion and Proton Conducting Electrolyte Materials for Clean Energy Applications: Structural and Mechanistic Features. Chem. Soc. Rev. 2010, 39 (11), 4370–4387. 10.1039/b915141a. 20848015

[ref11] Kwon O. H.; Choi G. M. Electrical Conductivity of Thick Film YSZ. Solid State Ion. 2006, 177 (35–36), 3057–3062. 10.1016/j.ssi.2006.07.039.

[ref12] Inaba H.; Tagawa H. Ceria-Based Solid Electrolytes. Solid State Ion. 1996, 83, 1–16. 10.1016/0167-2738(95)00229-4.

[ref13] Li M.; Pietrowski M. J.; De Souza R. A.; Zhang H.; Reaney I. M.; Cook S. N.; Kilner J. A.; Sinclair D. C. A Family of Oxide Ion Conductors Based on the Ferroelectric Perovskite Na_0.5_Bi_0.5_TiO_3_. Nat. Mater. 2014, 13 (1), 31–35. 10.1038/nmat3782. 24193663

[ref14] Majewski P.; Rozumek M.; Aldinger F. Phase Diagram Studies in the Systems La_2_O_3_-SrO-MgO-Ga_2_O_3_ at 1350–1400°C in Air with Emphasis on Sr and Mg Substituted LaGaO_3_. J. Alloys Compd. 2001, 329 (1–2), 253–258. 10.1016/S0925-8388(01)01583-3.

[ref15] Ishihara T.; Matsuda H.; Takita Y. Doped LaGaO_3_ Perovskite Type Oxide as a New Oxide Ionic Conductor. J. Am. Chem. Soc. 1994, 116 (9), 3801–3803. 10.1021/ja00088a016.

[ref16] Morales M.; Roa J. J.; Tartaj J.; Segarra M. A Review of Doped Lanthanum Gallates as Electrolytes for Intermediate Temperature Solid Oxides Fuel Cells: From Materials Processing to Electrical and Thermo-Mechanical Properties. J. Eur. Ceram. Soc. 2016, 36 (1), 1–16. 10.1016/j.jeurceramsoc.2015.09.025.

[ref17] Goodenough J. B.; Ruiz-Diaz J. E.; Zhen Y. S. Oxide-Ion Conduction in Ba_2_In_2_O_5_ and Ba_3_In_2_MO_8_ (M = Ce, Hf, or Zr). Solid State Ion. 1990, 44 (1–2), 21–31. 10.1016/0167-2738(90)90039-T.

[ref18] Fop S.; Skakle J. M. S.; McLaughlin A. C.; Connor P. A.; Irvine J. T. S.; Smith R. I.; Wildman E. J. Oxide Ion Conductivity in the Hexagonal Perovskite Derivative Ba_3_MoNbO_8.5_. J. Am. Chem. Soc. 2016, 138 (51), 16764–16769. 10.1021/jacs.6b10730. 27976879

[ref19] Fop S.; Mccombie K. S.; Wildman E. J.; Skakle J. M. S.; Irvine J. T. S.; Connor P. A.; Savaniu C.; Ritter C.; Mclaughlin A. C. High Oxide Ion and Proton Conductivity in a Disordered Hexagonal Perovskite. Nat. Mater. 2020, 19, 752–757. 10.1038/s41563-020-0629-4. 32123332

[ref20] Fop S.; Dawson J. A.; Tawse D. N.; Skellern M. G.; Skakle J. M. S.; McLaughlin A. C. Proton and Oxide Ion Conductivity in Palmierite Oxides. Chem. Mater. 2022, 34 (18), 8190–8197. 10.1021/acs.chemmater.2c01218. 36193291 PMC9523575

[ref21] Watson V.; Zhou Y.; Tawse D. N.; Ballantyne O. J.; Ridley C. J.; Dawson J. A.; Mclaughlin A. C. Tuning of the Ionic Conductivity of Ba_7_Nb_4_MoO_20_ by Pressure: A Neutron Diffraction and Atomistic Modelling Study. J. Mater. Chem. A Mater. 2025, 13, 4444–4451. 10.1039/D4TA08133A.

[ref22] Kendrick E.; Kendrick J.; Knight K. S.; Islam M. S.; Slater P. R. Cooperative Mechanisms of Fast-Ion Conduction in Gallium-Based Oxides with Tetrahedral Moieties. Nat. Mater. 2007, 6, 871–875. 10.1038/nmat2039. 17952081

[ref23] Waroquiers D.; Gonze X.; Rignanese G. M.; Welker-Nieuwoudt C.; Rosowski F.; Göbel M.; Schenk S.; Degelmann P.; André R.; Glaum R.; Hautier G. Statistical Analysis of Coordination Environments in Oxides. Chem. Mater. 2017, 29 (19), 8346–8360. 10.1021/acs.chemmater.7b02766.

[ref24] Jing X.-P.; West A. R. AC Impedance and Gas Concentration Cell Measurements for Ba_12_Y_4.67_Ti_8_O_35_. Acta Phys.-Chim. Sin. 2002, 18 (7), 617–623. 10.3866/PKU.WHXB20020710.

[ref25] Kuang X.; Allix M.; Ibberson R. M.; Claridge J. B.; Niu H.; Rosseinsky M. J. Oxygen Vacancy Ordering Phenomena in the Mixed-Conducting Hexagonal Perovskite Ba_7_Y_2_Mn_3_Ti_2_O_20_. Chem. Mater. 2007, 19 (11), 2884–2893. 10.1021/cm0626740.

[ref26] Mössner B.; Kemmler-Sack S. 9R-Stapelvarianten Vom Typ Ba_3_(B,B′)_2_O_9_-y Mit B,B′ ≡ Mo, W, V, Ti. J. Less-Common Met. 1985, 114 (2), 333–341. 10.1016/0022-5088(85)90453-9.

[ref27] Fop S.; McCombie K.; Smith R. I.; McLaughlin A. C. Enhanced Oxygen Ion Conductivity and Mechanistic Understanding in Ba_3_Nb_1-x_V_x_MoO_8.5_. Chem. Mater. 2020, 32, 4724–4733. 10.1021/acs.chemmater.0c01322.

[ref28] West A.; Irvine J.; Sinclair D. Electroceramics: Characterization by Impedance Spectroscopy. Adv. Mater. 1990, 2 (3), 132–138. 10.1002/adma.19900020304.

[ref29] Kuang X.; Green M. A.; Niu H.; Zajdel P.; Dickinson C.; Claridge J. B.; Jantsky L.; Rosseinsky M. J. Interstitial Oxide Ion Conductivity in the Layered Tetrahedral Network Melilite Structure. Nat. Mater. 2008, 7 (6), 498–504. 10.1038/nmat2201. 18488032

[ref30] Tate M. L.; Fuller C. A.; Avdeev M.; Brand H. E. A.; McIntyre G. J.; Evans I. R. Synthesis and Characterisation of New Bi(III)-Containing Apatite-Type Oxide Ion Conductors: The Influence of Lone Pairs. Dalton Trans. 2017, 46 (37), 12494–12499. 10.1039/C7DT02871G. 28895600

[ref31] Auckett J. E.; Lopez-Odriozola L.; Clark S. J.; Evans I. R. Exploring the Nature of the Fergusonite-Scheelite Phase Transition and Ionic Conductivity Enhancement by Mo^6+^ doping in LaNbO_4_. J. Mater. Chem. A 2021, 9 (7), 4091–4102. 10.1039/D0TA07453E.

[ref32] Gilane A.; Fop S.; Sher F.; Smith R.; Mclaughlin A. The Relationship between Oxide-Ion Conductivity and Cation Vacancy Order in the Hybrid Hexagonal Perovskite Ba_3_VWO_8.5_. J. Mater. Chem. A Mater. 2020, 8 (32), 16506–16514. 10.1039/D0TA05581F.

[ref33] Gilane A.; Fop S.; Tawse D. N.; Ritter C.; McLaughlin A. C. Variable Temperature Neutron Diffraction Study of the Oxide Ion Conductor Ba_3_VWO_8.5_. Inorg. Chem. 2022, 61 (3), 1597–1602. 10.1021/acs.inorgchem.1c03354. 35015549

[ref34] Momma K.; Ikeda T.; Belik A. A.; Izumi F. Dysnomia, a Computer Program for Maximum-Entropy Method (MEM) Analysis and Its Performance in the MEM-Based Pattern Fitting. Powder Diffr. 2013, 28 (3), 184–193. 10.1017/S088571561300002X.

[ref35] Sakata M.; Sato M. Accurate Structure Analysis by the Maximum-Entropy Method. Acta Crystallogr., Sect. A 1990, 46 (4), 263–270. 10.1107/S0108767389012377.

[ref36] Yashima M.; Enoki M.; Wakita T.; Ali R.; Matsushita Y.; Izumi F.; Ishihara T. Structural Disorder and Diffusional Pathway of Oxide Ions in a Doped Pr_2_NiO_4_-Based Mixed Conductor. J. Am. Chem. Soc. 2008, 130 (9), 2762–2763. 10.1021/ja711478h. 18257578

[ref37] Yashima M.; Tsujiguchi T.; Fujii K.; Niwa E.; Nishioka S.; Hester J. R.; Maeda K. Direct Evidence for Two-Dimensional Oxide-Ion Diffusion in the Hexagonal Perovskite-Related Oxide Ba_3_MoNbO_8.5-δ_. J. Mater. Chem. A 2019, 7 (23), 13910–13916. 10.1039/C9TA03588E.

[ref38] Fop S.; Dawson J. A.; Fortes A. D.; Ritter C.; McLaughlin A. C. Hydration and Ionic Conduction Mechanisms of Hexagonal Perovskite Derivatives. Chem. Mater. 2021, 33 (12), 4651–4660. 10.1021/acs.chemmater.1c01141.

[ref39] Meng J.; Sheikh M. S.; Jacobs R.; Liu J.; Nachlas W. O.; Li X.; Morgan D. Computational Discovery of Fast Interstitial Oxygen Conductors. Nat. Mater. 2024, 23 (9), 1252–1258. 10.1038/s41563-024-01919-8. 38871939

[ref40] Chance W. M.; Zur Loye H. C. Synthesis, Structure, and Optical Properties of a Series of Quaternary Oxides, K_2_Ba(MO_4_)_2_ (M = Cr, Mo, W). Solid State Sci. 2014, 28, 90–94. 10.1016/j.solidstatesciences.2013.12.013.

[ref41] Larson A. C.; Von Dreele R. B. General Structure Analysis System (GSAS); Los Alamos National Laboratory, 1994.

[ref42] Toby B. H. EXPGUI, a Graphical User Interface for GSAS. J. Appl. Crystallogr. 2001, 34 (1), 210–213. 10.1107/S0021889801002242.

[ref43] Tawse D. N.; Fop S.; Ritter C.; Martinez-Felipe A.; Mclaughlin A. C. A Variable Temperature Neutron Diffraction Study of Dual Ion Conducting Sr_3_V_2_O_8_. J. Solid State Chem. 2024, 331, 124512 10.1016/j.jssc.2023.124512.

[ref44] Kresse G.; Furthmüller J. Efficient Iterative Schemes for Ab Initio Total-Energy Calculations Using a Plane-Wave Basis Set. Phys. Rev. B 1996, 54 (16), 11169–11186. 10.1103/PhysRevB.54.11169. 9984901

[ref45] Kresse G.; Joubert D. From Ultrasoft Pseudopotentials to the Projector Augmented-Wave Method. Phys. Rev. B 1999, 59 (3), 1758–1775. 10.1103/PhysRevB.59.1758.

[ref46] Perdew J. P.; Ruzsinszky A.; Csonka G. I.; Vydrov O. A.; Scuseria G. E.; Constantin L. A.; Zhou X.; Burke K. Restoring the Density-Gradient Expansion for Exchange in Solids and Surfaces. Phys. Rev. Lett. 2008, 100 (13), 136406 10.1103/PhysRevLett.100.136406. 18517979

[ref47] Evans D. J.; Holian B. L. The Nose-Hoover Thermostat. J. Chem. Phys. 1985, 83 (8), 4069–4074. 10.1063/1.449071.

[ref48] Ong S. P.; Richards W. D.; Jain A.; Hautier G.; Kocher M.; Cholia S.; Gunter D.; Chevrier V. L.; Persson K. A.; Ceder G. Python Materials Genomics (Pymatgen): A Robust, Open-Source Python Library for Materials Analysis. Comput. Mater. Sci. 2013, 68, 314–319. 10.1016/j.commatsci.2012.10.028.

