# Supplementary material for: Unlocking the Potential of Palmierite Oxides: High Oxide Ion Conductivity via Induced Interstitial Defects
Source: J Am Chem Soc. 2025 Mar 7;147(11):9694–703. doi: 10.1021/jacs.4c17849 (PMC11926858; doi:10.1021/jacs.4c17849)
Supplement: Supplementary file 1 — ja4c17849_si_001.pdf [file ja4c17849_si_001.pdf]

Supplementary Information

for

Unlocking the potential of palmierite oxides: high oxide ion conductivity via induced interstitial defects

Dylan N. Tawse [a], Sacha Fop [a], John. W. Still [a], Oscar J. B. Ballantyne [b], Clemens Ritter [c], Ying Zhou [b], James A. Dawson [b] and Abbie C. Mclaughlin [a]

a Advanced Centre for Energy and Sustainability (ACES), The Chemistry Department, University of Aberdeen, Aberdeen AB24 3UE, United Kingdom

b Chemistry − School of Natural and Environmental Sciences, Newcastle University, Newcastle NE1 7RU, United Kingdom

c Institut Laue Langevin, 71 Avenue des Martyrs, BP 156, F-38042 Grenoble Cedex 9, France

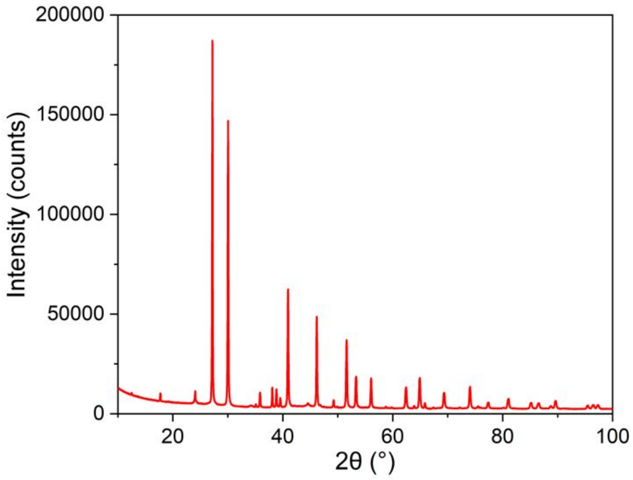

Figure S1. X-ray diffraction pattern of as-prepared $Ba_3Ti_{0.9}Mo_{1.1}O_{8.1}$. No impurity phases are present, and the pattern can be indexed with the space group $R\bar{3}m$ *H.*

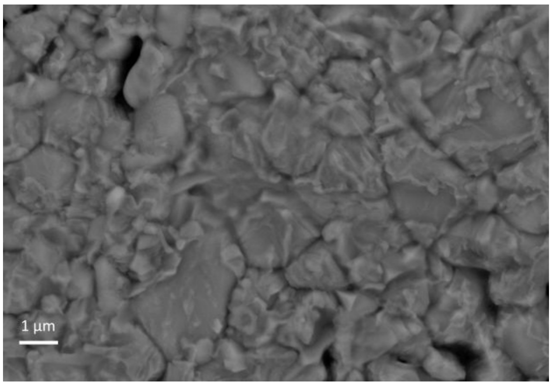

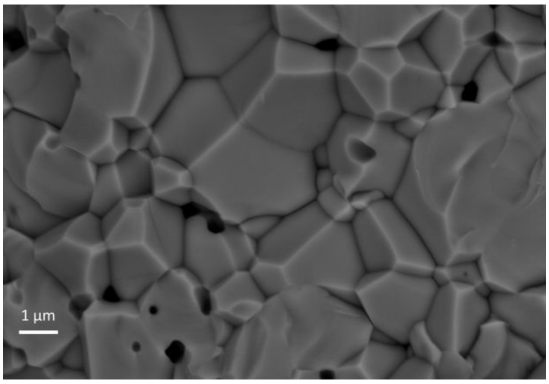

Figure S2 SEM micrograph of the surface (left) and section (right) of a 90% dense pellet of $Ba_3Ti_{0.9}Mo_{1.1}O_{8.1}$.

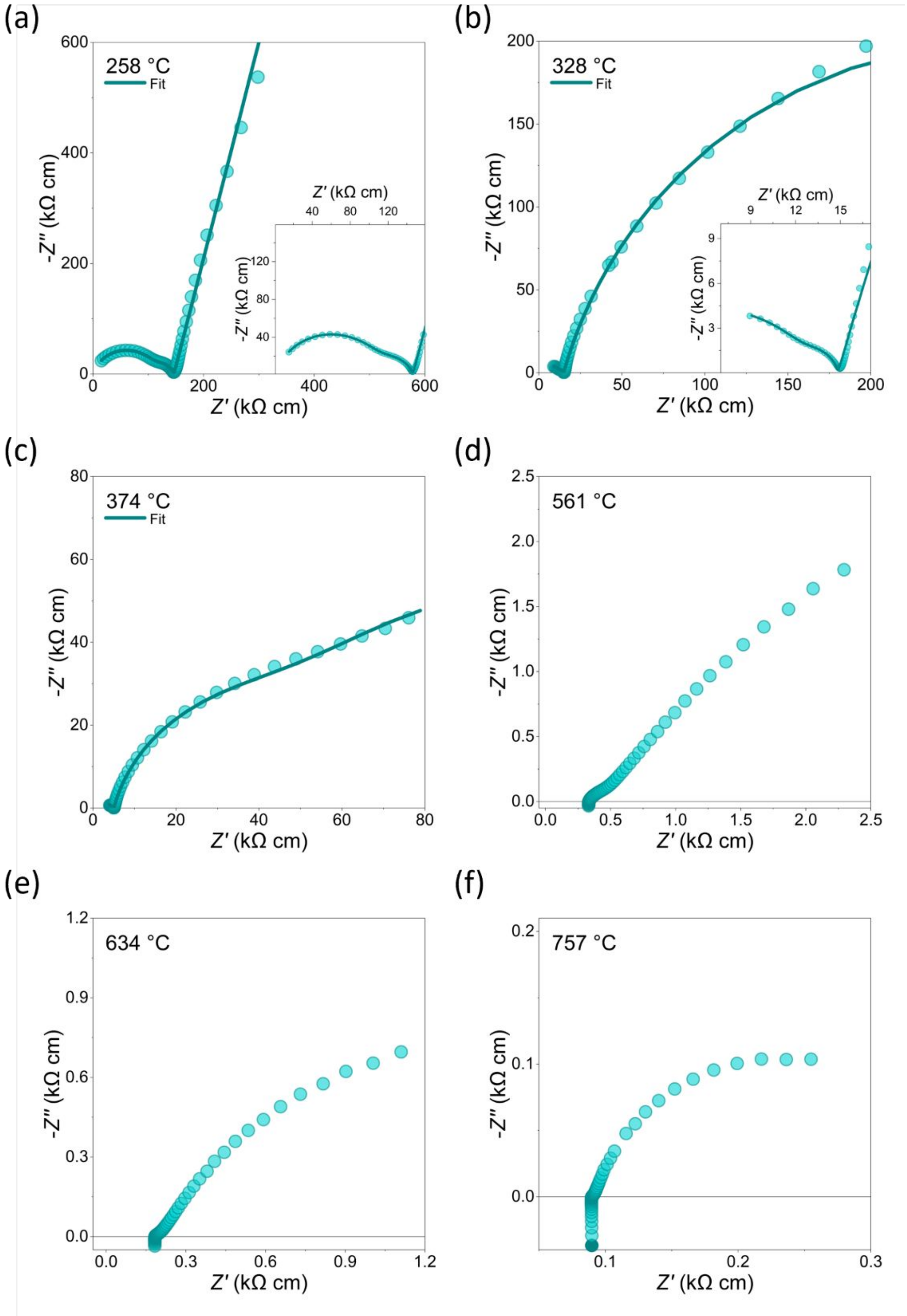

Figure S3. Complex impedance plots collected under dry air at various temperatures. Insets in (a) and (b) show magnification of high frequency regions. Equivalent circuit fits are shown for (a) – (c). The circles filled with a darker blue and corresponding numbers denote selected frequency decades in Hz.

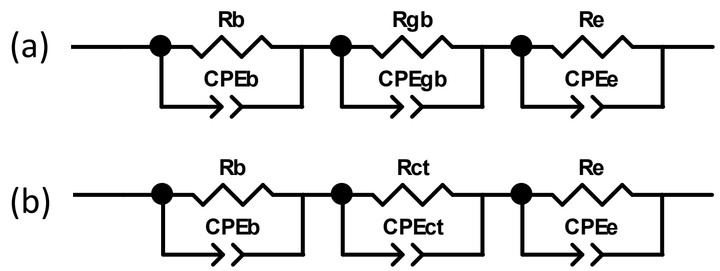

Figure S4. Equivalent circuit models used to fit impedance data. R represents a resistor while CPE represents a constant phase element. **(a)** Model composed of R in parallel with a CPE for the bulk (b), grain boundary (gb) and electrode (e) responses used to fit data from 162 - 352 °C. **(b)** Model composed of R in parallel with a CPE for the bulk (b), charge transfer region (ct) and electrode (e) responses used to fit data at 374 and 397 °C.

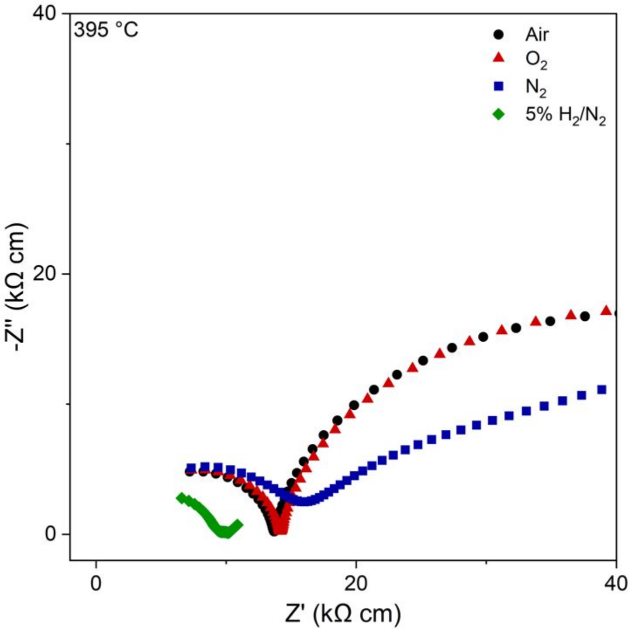

Figure S5. Complex impedance plot of $Ba_3Ti_{0.9}Mo_{1.1}O_{8.1}$ under dry air, O_2, N_2 and 5% H_2/N_2 at 395 °C. In the low frequency region under air, N_2 and O_2, the electrode response can be distinguished. The response is smaller under 5% H_2/N_2 but is still clearly identified.

The conductivity of the new sample measured under varying gaseous environments is slightly lower than the sample measured under dry air exclusively (1.02 x 10^{-3} S cm^{-1} (Figure 2 (d)) and 3.96 x 10^{-3} S cm^{-1} (Figure 2 (c)) under dry air at 600 °C, respectively). It is suspected that this may result from the slightly lower density of this sample, slight differences in oxygen stoichiometry, or microstructural changes resulting from the preparation of the sample for impedance measurements.

Table S1. Activation energies of $Ba_3Ti_{0.9}Mo_{1.1}O_{8.1}$ under various gases. Activation energies 1 and 2 are the E_a before and after the change in slopes observed above ~ 440 - 490 °C, respectively. The activation energies under air, O_2 and N_2 are comparable while those exhibited under 5% H_2/N_2 are higher than that observed under other gases.

Environment	Activation energy 1 (eV)	Activation energy 2 (eV)
**Dry air**	0.74(1)	0.52(1)
**Dry O_2**	0.76(1)	0.53(1)
**Dry N_2**	0.74(1)	0.52(2)
**Dry 5% H_2/N_2**	0.85(1)	0.65(0)

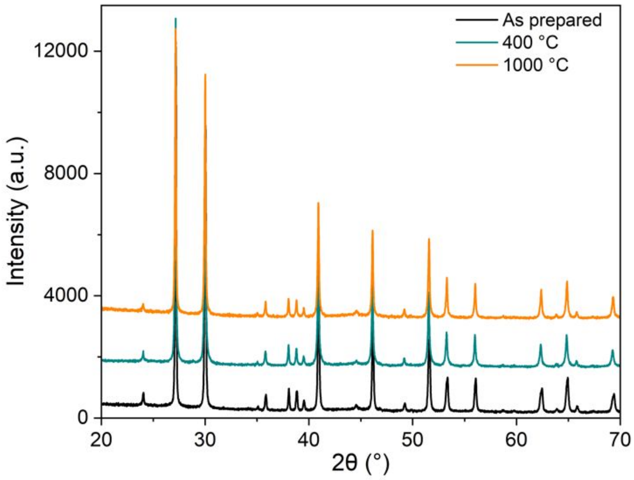

Figure S6. X-ray diffraction patterns of $Ba_3Ti_{0.9}Mo_{1.1}O_{8.1}$ after exposure to dry air at 400 °C and 1000 °C. No impurity phases were observed in the temperature range 1000 to 400 °C.

(a)

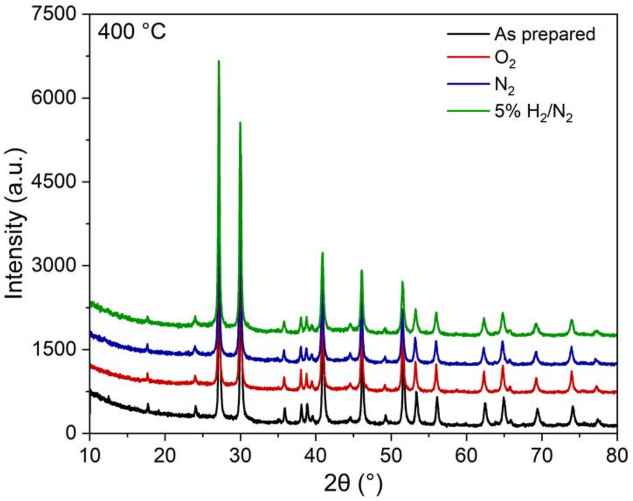

(b)

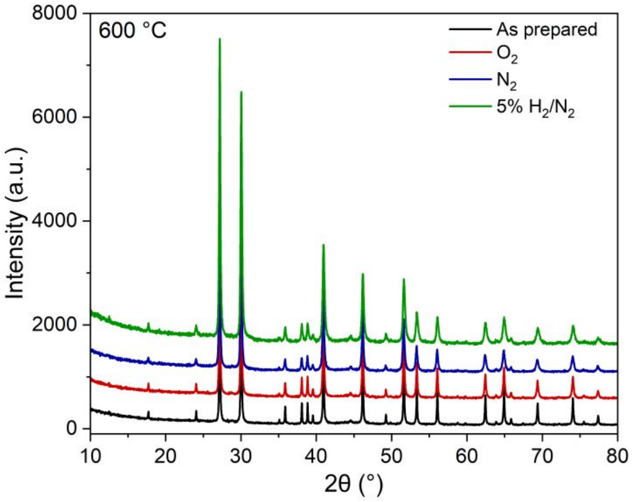

Figure S7. X-ray diffraction patterns of $Ba_3Ti_{0.9}Mo_{1.1}O_{8.1}$ post annealing in O_2, N_2 and 5% H_2/N_2 at (a) 400 °C and (b) 600 °C. No impurity phases were detected under these conditions at either temperature.

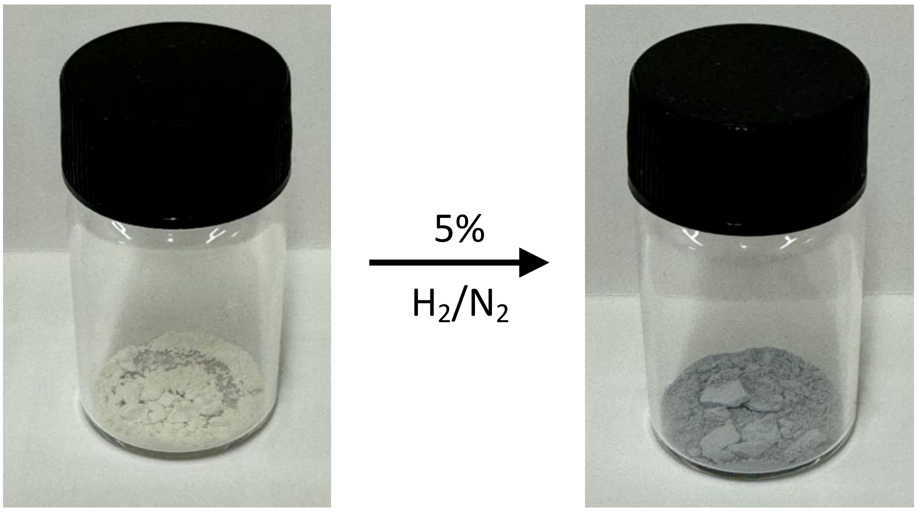

Figure S8. $Ba_3Ti_{0.9}Mo_{1.1}O_{8.1}$ before and after exposure to 5% H_2/N_2. A color change from off white to blue is observed. This color change is likely produced by the reduction of Ti^{4+} to Ti^{3+} and/or Mo^{6+} to Mo^{5+}.

TGA-MS was performed to determine mass loss upon heating of the sample and the species lost from 25 to 1000 °C. Approximately 0.5 mg was lost upon heating (Figure S8 (a)). A small amount of H_2O was released below 600 °C. The greatest mass loss is attributed to CO_2 which is mostly expelled between 600 to 900 °C (Figure S8 (b)). The water loss would primarily be linked to the release of surface water below 200 °C with a small amount of absorbed water released above 200 °C. The total mass losses correspond to $n = 8.83 \times 10^{-5}$ molecules of H_2O and $x = 0.098$ molecules of CO_2 per formula unit for $Ba_3Ti_{0.9}Mo_{1.1}O_{8.1}{\bullet}nH_2O{\bullet}xCO_2$.

(a)

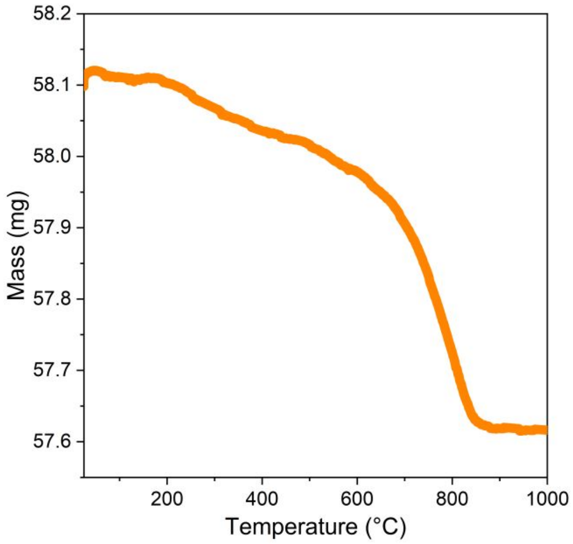

(b)

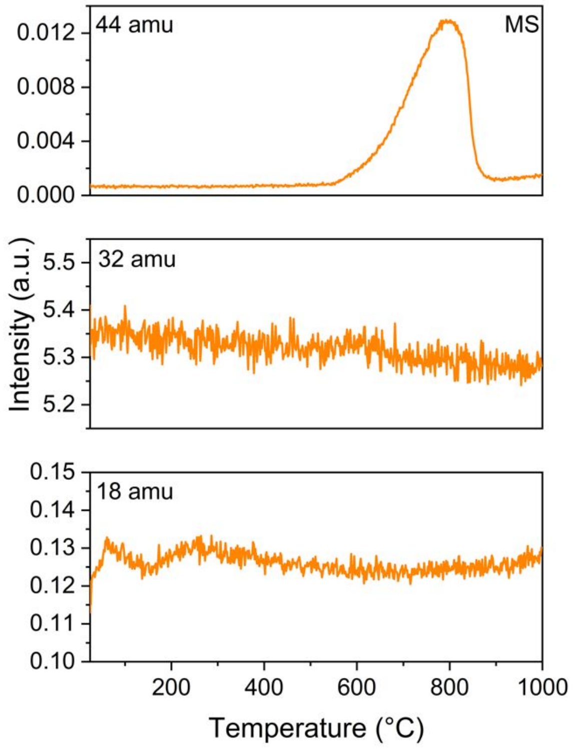

Figure S9. TGA-MS analysis performed under dry air on a freshly prepared sample after a 2 hour pre-heat at 300 °C to remove surface species. (a) Mass loss (in mg) observed upon heating from 25 to 1000 °C. (b) Mass spectrometry results were obtained at 44 amu (CO_2), 32 amu (O_2) and 18 amu (H_2O).

Table S2. Refined atomic parameters from Rietveld fit of variable temperature neutron diffraction data of $Ba_3Ti_{0.9}Mo_{1.1}O_{8.1}$ collected on the D2B diffractometer at 25 °C and 700 °C. U_{ij} (in Å^2) represent anisotropic displacement parameters. Where occupancies refined to within ±1% of full occupancy, they were fixed at 1 and therefore have been omitted from this table.

			**25 °C**	**700 °C**
**Ba1**	**(0,0,0)**	$U_{11} = U_{22}$	0.071(2)	0.106(3)
	3a	U_{33}	0.003(2)	0.030(3)
		U_{12}	0.036(1)	0.053(2)
**Ba2**	**(0,0,*z*)**	*z*	0.2028(1)	0.2030(2)
	**6*c***	$U_{11} = U_{22}$	0.0220(7)	0.054(1)
		U_{33}	0.014(1)	0.026(2)
		U_{12}	0.0110(4)	0.0269(7)
**Mo**	**(0,0,*z*)**	*z*	0.3970(2)	0.3971(3)
	**6*c***	$U_{11} = U_{22}$	0.005(1)	0.025(1)
		U_{33}	0.005(1)	0.025(1)
		U_{12}	0.0026(7)	0.0125(7)
		Fraction	0.552(3)	0.552(3)
**Ti**	**(0,0,*z*)**	*z*	0.3970(2)	0.3971(3)
	**6*c***	$U_{11} = U_{22}$	0.005(1)	0.025(1)
		U_{33}	0.005(1)	0.025(1)
		U_{12}	0.0026(7)	0.0125(7)
		Fraction	0.448(3)	0.448(3)

Table S2. Continued.

			25 °C	700 °C
**O1**	**(x,y,*z*)**	***x***	0.010(2)	0.062(1)
	**36*i***	***y***	0.0525(9)	0.003(2)
		z	0.3231(1)	0.3243(2)
		U_{iso}	0.016(1)	0.043(2)
		Fraction	0.158(1)	0.160(3)
**O2**	**(x,y,*z*)**	***x***	0.1700(1)	0.1716(2)
	**18*h***	***y***	0.8230(1)	0.8284(2)
		z	0.10123(6)	0.10066(8)
		$U_{11} = U_{22}$	0.0222(4)	0.0624(9)
		U_{33}	0.0269(6)	0.061(1)
		U_{12}	0.0173(6)	0.047(1)
		U_{13}	-0.0001(4)	0.0020(6)
		U_{23}	0.0001(4)	-0.0020(6)
**O3**	**(x,y,*z*)**	***x***	0.541(10)	0.526(10)
	**36*i***	***y***	0.591(8)	0.606(8)
		z	0.003(4)	0.006(3)
		U_{iso}	0.05(2)	0.03(1)
		Fraction	0.017(1)	0.015(3)

Table S2. Continued.

	**25 °C**	**700 °C**
***a* (Å)**	5.95403(7)	6.0336(1)
***c* (Å)**	21.2591(4)	21.4017(5)
***V* ($Å^3$)**	652.68(2)	674.73(4)
**χ^2**	1.857	1.522
**R_p (%)**	2.18	1.92
**R_{wp} (%)**	2.82	2.45

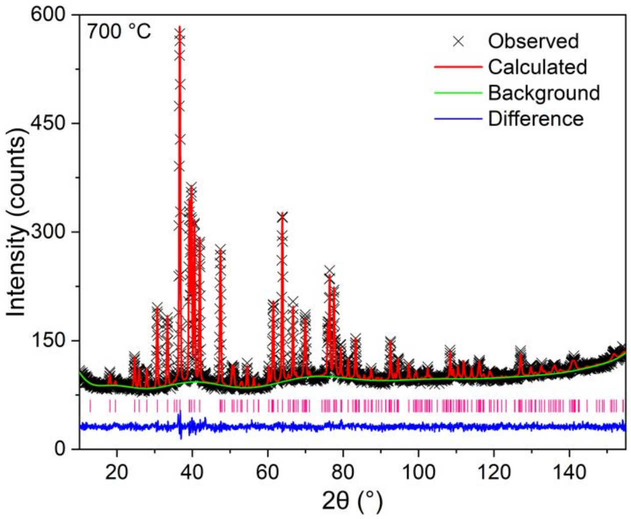

Figure S10. Fitted neutron diffraction histogram for $Ba_3Ti_{0.9}Mo_{1.1}O_{8.1}$ collected on D2B at 700 °C. Black crosses show observed data, red line the Rietveld fit, blue line the difference between the observed and calculated patterns, green line the background function, and the pink vertical bars show the reflection positions.

Table S3. Selected bond lengths (in Å) and bond angles (in °) calculated by Rietveld refinement of powder neutron diffraction data of $Ba_3Ti_{0.9}Mo_{1.1}O_{8.1}$ at 25 and 700 °C. M represents Mo/Ti. Large errors are displayed for the O2-M-O3 angles due to the split site O3 occupies and large static disorder.

**Bond length (Å)**	**25 °C**	**700 °C**
**Ba1-O2**	2.776(1)	2.804(2)
**Ba1-O3**	2.60(4)	2.66(5)
**Ba2-O1**	2.574(4)	2.623(4)
**Ba2-O2**	2.781(3)	2.831(3)
	3.0419(6)	3.0831(8)
**M-O1**	1.597(5)	1.598(8)
**M-O2**	1.864(2)	1.866(3)
**M-O3**	2.00(7)	1.84(5)
**Bond angle (°)**		
**O1-M-O2**	107.2(3)	103.2(3)
	113.2(4)	115.0(4)
	125.1(2)	126.0(4)
**O2-M-O2**	103.0(2)	103.3(2)
**O2-M-O3**	78(2)	78(2)
	96(2)	97(1)
	160(2)	159(2)

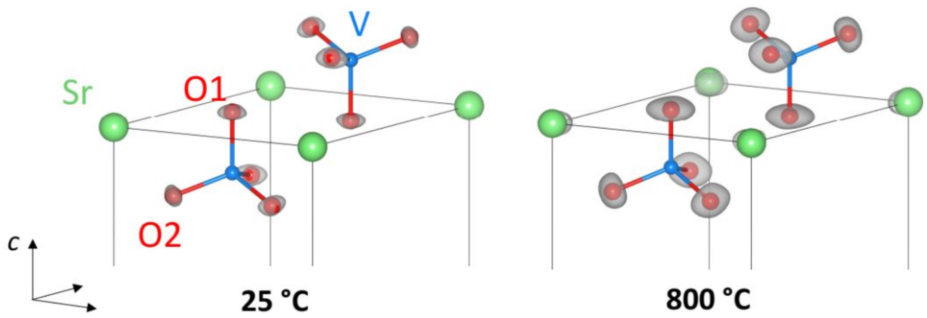

Figure S11. Nuclear scattering density distribution for $Sr_3V_2O_8$ at 25 °C and 800 °C reconstructed via maximum entropy (MEM) analysis. The neutron data at 800 °C does not show any oxide ion connectivity on the [SrO_2] plane.

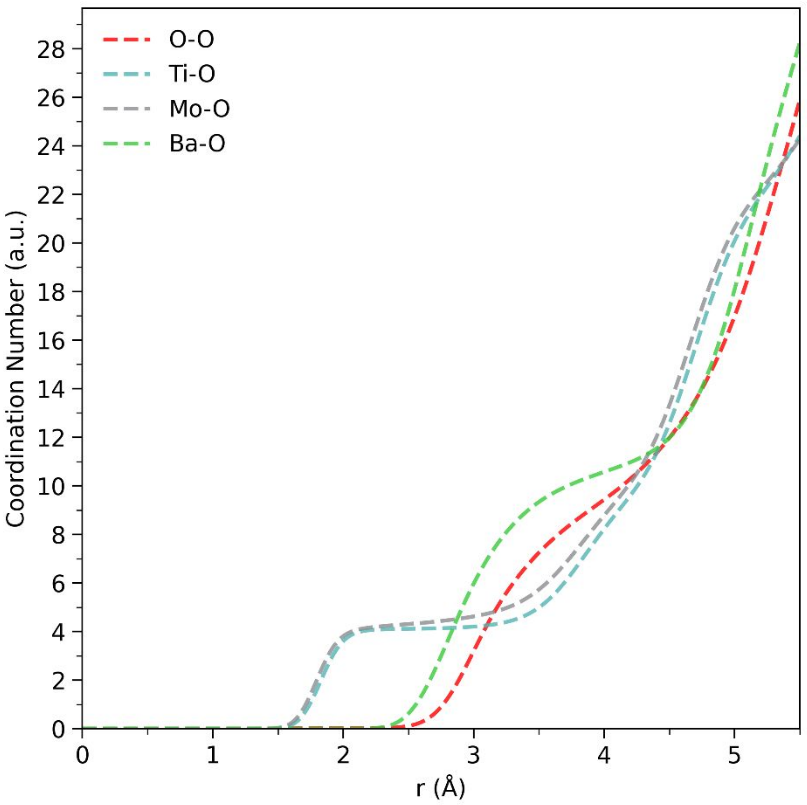

Figure S12. Integrated RDFs of O-O, Ti-O, Mo-O, and Ba-O pairs at 850 K.

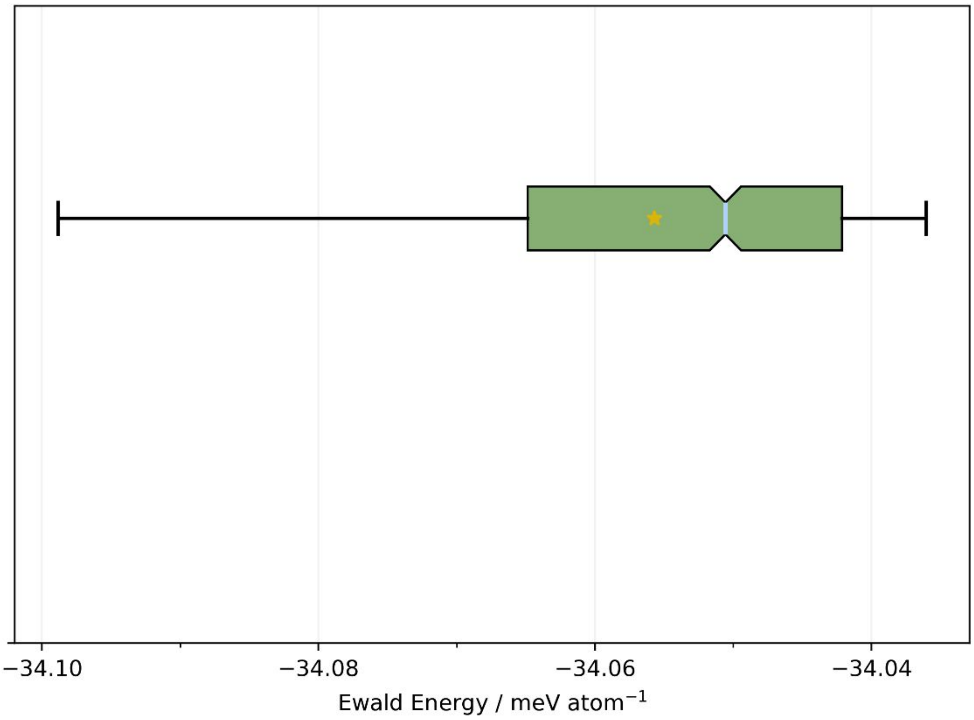

Figure S13. Ewald energy per atom distribution of 1000 structural conformations of $Ba_3Ti_{0.9}Mo_{1.1}O_{8.1}$. Black whiskers represent range of energies, two sections of the green box represent lower and upper quartile ranges, respectively. The gold star represents the mean value and the light blue line represents the median.
